# Enhancing detection of topological order by local error correction

**DOI:** 10.1038/s41467-024-45584-6

**Published:** 2024-02-20

**Authors:** Iris Cong, Nishad Maskara, Minh C. Tran, Hannes Pichler, Giulia Semeghini, Susanne F. Yelin, Soonwon Choi, Mikhail D. Lukin

**Affiliations:** 1 Department of Physics, Harvard University, Cambridge, MA 02138 USA; 2 Center for Theoretical Physics, Massachusetts Institute of Technology, Cambridge, MA 02139 USA; 3 Institute for Theoretical Physics, University of Innsbruck, 6020 Innsbruck, Austria; 4 Institute for Quantum Optics and Quantum Information of the Austrian Academy of Sciences, 6020 Innsbruck, Austria

**Keywords:** Quantum information, Quantum simulation

## Abstract

The exploration of topologically-ordered states of matter is a long-standing goal at the interface of several subfields of the physical sciences. Such states feature intriguing physical properties such as long-range entanglement, emergent gauge fields and non-local correlations, and can aid in realization of scalable fault-tolerant quantum computation. However, these same features also make creation, detection, and characterization of topologically-ordered states particularly challenging. Motivated by recent experimental demonstrations, we introduce a paradigm for quantifying topological states—locally error-corrected decoration (LED)—by combining methods of error correction with ideas of renormalization-group flow. Our approach allows for efficient and robust identification of topological order, and is applicable in the presence of incoherent noise sources, making it particularly suitable for realistic experiments. We demonstrate the power of LED using numerical simulations of the toric code under a variety of perturbations. We subsequently apply it to an experimental realization, providing new insights into a quantum spin liquid created on a Rydberg-atom simulator. Finally, we extend LED to generic topological phases, including those with non-abelian order.

## Abstract (web summary)

Detection of topological phases in experiments is challenging, especially in the presence of incoherent noise. Cong et al. introduce a novel method combining error correction and renormalization-group flow and apply it to characterization of quantum spin liquid phases realized in a Rydberg-atom simulator.

## Introduction

Topological ordering is an exotic phenomenon that can occur when quantum fluctuations and local constraints stabilize a state with long-range entanglement^1^. With their non-local correlations, topologically ordered states feature many remarkable properties and can be used for protecting quantum information non-locally^1–3^. Yet, because these states appear to be liquid-like at short length-scales^4^, they cannot be identified or characterized using any local order parameters. Instead, the canonical approach to discern topological order is to measure operators supported on large closed loops, the Wilson loops^1,5–7^. However, such operators are challenging to identify or measure: while they have simple forms in certain fixed-point models, this is generally not the case for states realized experimentally in the presence of noise or other perturbations. In these cases, the expectation values of the simple or ‘bare’ Wilson loop operators described above decay exponentially with the loop’s perimeter, which hinders the experimental certification of topological order.

To address these challenges, several methods have been developed to construct ‘fattened’ Wilson loops which do not decay with loop size. These include a systematic method utilizing quasi-adiabatic connections to the fixed-point models^5^, as well as variational and tensor-network-based approaches^8–11^. Nevertheless, these methods are challenging to apply in realistic experiments, especially in the presence of incoherent noise (e.g., spontaneous emission). Other signatures, such as topological entanglement entropy^12,13^ are likewise difficult to measure in large systems.

Motivated by these considerations, we introduce a powerful framework, *locally error-corrected decoration (LED)*, for studying and characterizing topologically ordered states. By leveraging the error-correcting properties of topological phases, LED provides a systematic method to construct and efficiently measure ‘decorated’ Wilson loop operators, a variant of the fattened loop operators. This enables the identification and characterization of topological order at large length-scales in the presence of both coherent perturbations and incoherent noise, which are particularly challenging or impossible using conventional methods.

In its most general form, LED corresponds to a class of hierarchically structured quantum circuits that resemble the classification of quantum phases using RG flow^14,15^. Yet, for a wide range of experiments where the prepared state is known to approximate a fixed-point state with zero correlation length (see Supplementary Information), there is an efficient ‘snapshot-based’ realization of LED using only classical post-processing of experimental measurements in a few fixed bases. In this work, we primarily focus on snapshot-based LED due to current experimental limitations and the hardness of simulating 2D quantum circuits.

## Results

### LED approach

The key idea of LED can be understood by considering Kitaev’s toric code model, a canonical example of topological order. Specifically, we consider qubits localized on the edges of a square lattice. The ideal, fixed-point Hamiltonian is defined as^16^:

1 $${H}_{{{{{{{{\rm{TC}}}}}}}}}=-J\mathop{\sum}\limits_{v}{A}_{v}-J\mathop{\sum}\limits_{p}{B}_{p}$$

where *A*_*v*_ = ∏_*i*∈adj(*v*)_*X*_*i*_, *B*_*p*_ = ∏_*i*∈adj(*p*)_*Z*_*i*_, and adj(*v*) (resp., adj(*p*)) denote the set of edges touching a given vertex *v* (plaquette *p*) of the lattice. The ground state space, given by the simultaneous + 1 eigenspace of all *stabilizer operators* {*A*_*v*_, *B*_*p*_}, forms a quantum error-correcting code: all local operators either act trivially on ground states or couple them to excited states^16^. By measuring stabilizers, one can detect the presence of excitations and apply a recovery procedure to return the system back to this ground state space.

**Fig. 1:**
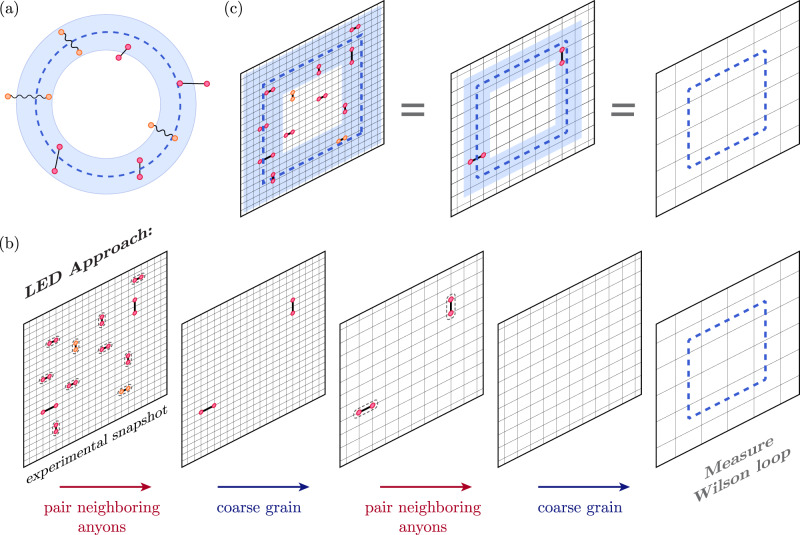
Detecting topological phases via snapshot-based LED. **a** In the absence of perturbations, a topologically ordered state with zero correlation length such as Kitaev’s toric code state^16^ is characterized by +1 expectation values of `bare' Wilson loop operators, which are typically tensor products of single-site operators (dotted blue loop). In realistic systems, however, coherent perturbations give rise to virtual anyon pairs (red dots/straight lines), and incoherent errors introduce physical anyon pairs (orange dots/wavy lines); this causes the expectation value of bare Wilson operators to decay exponentially with the loop’s perimeter. To account for these local fluctuations, one can measure `fattened' Wilson operators supported on an annulus (blue); the LED loops constitute one realization of this. **b** LED method to measure decorated Wilson loop observables for ${{\mathbb{Z}}}_{2}$ topological order in a system where qubits live on the links of a square lattice, and stabilizers are associated with vertices. Given an experimental snapshot of all qubits in the *Z* or *X* basis, one can obtain values for all stabilizer operators in that basis, thereby identifying the locations of all *e* or *m* anyons, respectively. In the first step, neighboring anyons are paired using a local decoder (dashed pairings), and each pair is removed by flipping the value(s) of qubit(s) lying on a path of minimal length connecting the two anyons; subsequently, the lattice is coarse-grained so that only a fraction of the original qubits remain. These two steps are iterated *n* times (here, *n* = 2), after which a bare Wilson loop is evaluated on the final, coarse-grained state. **c** The final, bare Wilson loop operator evaluated on the final state is equivalent to decorated Wilson loop operators evaluated at earlier iterations (see Methods).

In this model, contractible Wilson loops can be constructed by multiplying stabilizers, so their expectation values in any ground state of *H*_TC_ are +1, independent of loop size. However, in realistic situations, the prepared state differs from the fixed-point state by local fluctuations such as coherent perturbations and incoherent errors (Fig. 1a). This causes bare Wilson loops to decay exponentially with the number of locations where a fluctuation can intersect the loop (i.e., its perimeter).

The snapshot-based LED approach begins with a measurement of all qubits in the same (Pauli-*Z* or Pauli-*X*) basis. For each measurement snapshot, one can calculate the stabilizer and Wilson loop values. Local fluctuations appear as stabilizer violations, which are identified with anyonic excitations^16^ (Fig. 1b). A local decoder partially removes such fluctuations by flipping measured qubits using only nearby stabilizer values. The simplest such local decoder can remove single-qubit errors, by flipping a qubit if and only if both adjacent vertices (resp., plaquettes) are occupied by an *m* (*e*-anyon). However, it cannot remove higher-weight errors, which flip two or more adjacent qubits. Subsequently, the lattice is coarse-grained, which can also be done efficiently on measurement snapshots (see methods). Together, the anyon-pairing and coarse-graining steps are repeated for *n* layers. Crucially, the weight of uncorrected errors is reduced in each layer, so that all local errors eventually become single-qubit errors, which the decoder can correct; this mimics a real-space RG flow towards the fluctuation-free fixed-point state (see Methods). Finally, a bare Wilson loop is measured on the final, corrected and coarse-grained state (Fig. 1b).

This bare operator measured on the final state is equivalent to a decorated Wilson loop operator measured on the original state (Fig. 1). Moreover, it is determined solely by the fixed-point state and is independent of the specific fluctuations in the system; this crucially differentiates LED from prior approaches to construct fattened loop operators^5,8,9^. Notice that all steps in snapshot-based LED can be performed in post-processing (see Methods), making it uniquely suited for integration into experimental measurement procedures. More general LED operators can be constructed through the quantum circuit formulation; one example is presented in the Supplementary Information.

**Fig. 2:**
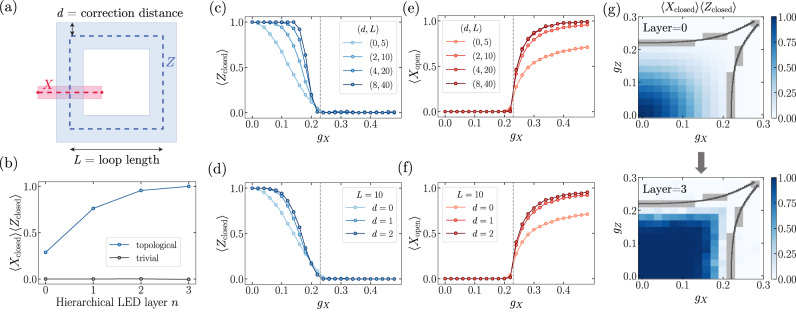
Numerical demonstration with coherently perturbed toric code states. **a** In a general construction of our LED Wilson loop operator, we use a local decoder which pairs anyons within a region of radius *d* (blue annulus). Conjugate LED open string operators (red stripe) anti-commute with Wilson loops, and hence must vanish in the topological phase. **b** Order parameter 〈*X*_loop_〉〈*Z*_loop_〉 for a trivial state (*g*_*Z*_ = 0.0, *g*_*X*_ = 0.26) and a topological state (*g*_*Z*_ = 0.12, *g*_*X*_ = 0.12), upon varying *n*, using a distance-four patch decoder and coarse-graining blocksize two respectively (see Methods). **c** Output at different *n* along the *g*_*Z*_ = 0.14 line of the phase diagram. Gray dotted line is conjectured phase transition region. **d** Expectation values of generic LED Wilson loops with the same diameter *L*, using the pairing decoder (*d* = 1) and distance-four patch decoder (*d* = 2) without coarse-graining. **e**, **f** Corresponding expectation values of bare and decorated open string operators. **g** Order parameter values constructed from bare Wilson loops (*n* = 0) and LED Wilson loops (*n* = 3), using the same LED procedure as (**c**, **e**), across varying values of (*g*_*X*_, *g*_*Z*_). Dark gray regions are numerical estimates for the phase boundary between topological and trivial (Methods). Light gray regions correspond to locations where sampling is expensive due to large correlation length.

The hierarchical LED procedure is also inspired by the quantum convolutional neural network (QCNN) approach to phase classification, and the decorated Wilson loop operators resemble the multiscale string order parameters studied in ref. ^17^. However, in this context, the LED framework is more general: one can construct LED Wilson operators of diameter *L* with any desired correction distance *d* ≪ *L* (Fig. 2a) by choosing any local decoder which pairs anyons up to distance *d* (see Methods). The construction of Fig. 1b with alternating local-decoding and coarse-graining layers is a particularly efficient way to construct local decoders and LED loops with longer-range (e.g., *d*, *L* ∝ 2^*n*^).

We emphasize that the locality of our procedure ensures that only topologically ordered states can flow to the fixed-point state. Thus, LED gives rise to a sufficient condition or *witness* for topological order. This distinguishes LED from general decoders, which do not typically respect locality and hence cannot be used to certify topological order.

### Numerical detection of topological order with coherent perturbations

To demonstrate the applicability of LED for coherent local perturbations to *H*_TC_, we consider a family of states

2 $$\left| \psi ({g}_{X},\, {g}_{Z})\right\rangle=\frac{1}{{{{{{{{\mathcal{N}}}}}}}}}{e}^{{g}_{X}\mathop{\sum}\limits_{i}{X}_{i}+{g}_{Z}\mathop{\sum}\limits_{i}{Z}_{i}}\left| {\psi }_{{{{{{{{\rm{TC}}}}}}}}}\right\rangle,$$

generated by imaginary-time evolution of a toric code ground state $\left\vert {\psi }_{{{{{{{{\rm{TC}}}}}}}}}\right\rangle$^18–20^. As each operator *Z*_*i*_ (resp., *X*_*i*_) creates a pair of *m* anyons (*e* anyons), $\left\vert \psi ({g}_{X},\, {g}_{Z})\right\rangle$ contains virtual anyon fluctuations on top of $\left\vert {\psi }_{{{{{{{{\rm{TC}}}}}}}}}\right\rangle$. In the special case where *g*_*X*_ = 0, topological order is known to survive for perturbations *g*_*Z*_ ≤ *g*_*c*_ = 0.220343, beyond which the *m*-anyons condense, driving a second-order phase transition into the *Z*-paramagnet state^21^. More generally, $\left\vert \psi ({g}_{X}\ne \, 0,{g}_{Z}\ne \, 0)\right\rangle$ is also topologically ordered for small *g*_*X*_ and *g*_*Z*_, but the transitions to paramagnetic phases can occur at points that differ from *g*_*c*_.

In testing LED, we numerically simulate projective measurements of $\left\vert \psi ({g}_{X},\, {g}_{Z})\right\rangle$ and use them as input “experimental snapshots” in Fig. 1b (see Methods). Figure 2b shows the value of the LED order parameter for a trivial and a topological state with *g*_*Z*_ = 0.14, when *n* is varied (and *d*, *L* ∝ 2^*n*^). Clearly, the order parameter stays at 0 for the trivial state, but increases from a small, finite value to one for the topological state as *n* is increased. Similar behavior is also observed throughout a one-dimensional parameter space in Fig. 2c, d, whenever the correction distance *d* is increased, while keeping *d* ≪ *L* to prevent overcorrection (see Methods). Importantly, amplification occurs only if the input state is topological, and the order parameter approaches 0 for all trivial states.

Another important set of observables for characterizing topological order are *X* and *Z* open string operators, which detect the transition from the topological phase to the trivial, paramagnet phase. Because LED Wilson *Z*-loop operators (resp., *X*-loop operators) are linear combinations of *Z* (*X*) closed loops supported on an annulus, they anti-commute with conjugate *X* (*Z*) open strings connecting the interior and exterior of the annulus. As such, the expectation value of any long, open string must flow to zero in the topological phase, whereas closed-loop LED operators flow to unity with increasing *d*. The topological-to-trivial phase transition occurs when certain long, open *X* or *Z* strings acquire non-zero expectation value, due to the condensation of *m* or *e* anyons, respectively. Indeed, deep in the paramagnetic phase the state $\mathop{\lim }\limits_{{g}_{x}\to \infty }\left\vert ({g}_{x},{g}_{z})\right\rangle$ is polarized along the *X* direction, and *X* open strings become unity. However, for generic trivial states, open strings also decay exponentially with length, due to local fluctuations of the opposite type; nevertheless, LED can still amplify their expectation values by removing the effect of local fluctuations. This behavior is demonstrated in our simulations: in Fig. 2e, f, open string expectation values stay at 0 in the topological phase, but are amplified and saturate to a non-zero value in the trivial (paramagnetic) phase. Because LED amplifies the contrast between trivial states and a large class of topological states, the topological order can be detected using with lower sample complexity—that is, by using substantially fewer experimental repetitions^17,22^ (see Supplementary Information).

Let us note that the boundary dividing the states whose LED operators approach zero and one does not necessarily correspond to the topological phase boundary: in general, it depends on the choice of decoder and coarse-graining length-scale. For instance, this is observed in Fig. 2g, where closed loops are nearly one after *n* = 3 layers for a large region within, but not fully encompassing, the topological phase. Hence, LED is not always a necessary condition for topological order.

### Effect of incoherent errors

We next demonstrate the application of LED in the presence of incoherent local noise such as spontaneous emission or dephasing, which commonly occur in experiments. Because local decoders can recover topologically encoded information in the presence of small, local error channels^12,23^ it is reasonable to ask whether mixed states prepared in these systems exhibit topological ordering.

**Fig. 3:**
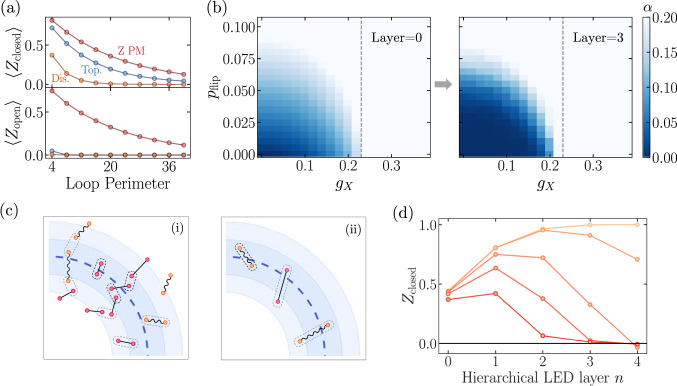
Application to mixed states. **a** Without error correction, generic points in the topological and trivial, disordered phase (*g*_*Z*_ = 0.12, *g*_*X*_ = 0.18, *p*_flip_ = 0.0 and *g*_*Z*_ = 0.06, *g*_*X*_ = 0.0, *p*_flip_ = 0.11 resp. shown in the plot) appear very similar qualitatively, as closed loops decay exponentially with loop perimeter in both cases, while open strings remain close to zero (see Methods). In contrast, in the trivial, paramagnet phase (*g*_*Z*_ = 0.32, *g*_*X*_ = 0.2, *p*_flip_ = 0), open strings decay with the same perimeter-law as closed loops. **b** *g*_*Z*_ = 0.14 slice of mixed state phase diagram, containing topological, disordered, and *X*-paramagnetic phases. These phases are associated with fixed-point states *g*_*x*_7D2 = 7D2*g*_*z*_7D2 = 7D2*p*_flip_7D2 = 7D20, *g*_*z*_7D2 → 7D2*∞*, *g*_*x*_7D2 → 7D2*∞*, and *p*_flip_7D2 → 7D20.5, respectively. The flow of the closed-loop decay exponent *α* under LED provides a sharp divider between two kinds of perimeter-law decay, observed in different regimes of the mixed-state phase diagram. **c** In the uncorrectable regime (i), the local decoder of LED pairs anyons incorrectly, resulting in perimeter-law decay with large *α* in disordered and paramagnetic phases. Moreover, the probability of such an incorrect pairing can increase with the number *n* of LED iterations. Here, the black pairings are made by LED at or before one specific value of *n*, and gray pairings are made upon performing one additional LED iteration. In the correctable (topological) regime (ii), increasing *n* can reduce *α* to zero, as fluctuations of higher characteristic length *ξ* can be reliably corrected using only local information. In the conceptual framework where an LED operator is embedded in a surface code on an annulus (Fig. 2a), incorrect pairings corresponds to logical errors (e.g. *X*_*L*_). **d** Expectation values of LED loop observables upon increasing *n* (*d*, *L* ∝ 2^*n*^), in thermal states of varying temperatures (between 0 and 0.35, with darker colors indicating higher temperatures) and *p*_flip_ = 0.02.

To study such examples, we introduce incoherent bit- and phase-flip errors by independently flipping, with probability *p*_flip_, each measured qubit in a snapshot of $\left\vert \psi ({g}_{X},{g}_{Z})\right\rangle$. Here, we associate topological order with states that can be transformed into a ground state of *H*_TC_ via local operations. Our analysis then suggests that the resulting mixed-state phase space contains a ${{\mathbb{Z}}}_{2}$-topological phase, a *Z*-paramagnet, an *X*-paramagnet, and a disordered phase with large incoherent error rates. However, it is especially difficult to distinguish the topological and disordered phases using measurements of bare operators alone: in both phases, open strings remain close to zero, while bare Wilson loops decay exponentially with perimeter as *e*^−*αL*^, where the exponent *α* interpolates smoothly between the phases (Fig. 3a, b). This is in contrast to the paramagnet phases, where closed loops exhibit similar behavior, but certain open strings decay with the same exponent *α* as the closed loops^24^.

Upon studying the behavior of LED operators, one finds that the mixed-state phase space exhibits two qualitatively different regimes (Fig. 3b). LED reduces *α* to 0 with increasing *d* in the ‘correctable’ regime, while *α* grows in the ‘uncorrectable’ regime. Further, correctable states with small *p*_flip_ are connected to topologically ordered pure states, suggesting these mixed states are topologically ordered as well. Indeed, we show that correctability implies the input state cannot be prepared from a product state using only local operations. In particular, if LED Wilson loops are amplified to above 1 − *ϵ* under depth *d* correction, this certifies topological order up to length-scale $O({{{{{{{\mathscr{L}}}}}}}}-d)$ where ${{{{{{{\mathscr{L}}}}}}}} \sim 1/\sqrt{\epsilon }$. Furthermore, we argue (see Methods) that, under plausible conditions, this implies the *entanglement negativity* of the input state contains a topological term; this connects the LED characterization of mixed state topological order to other studies^25–27^.

The ability of LED to distinguish between the topological and disordered phases can be understood by analogy to quantum error correction. Conceptually, since any given LED loop operator is supported on an annulus, we can consider this operator as being embedded in a surface code on this annulus with open boundary conditions, which supports a logical qubit. Then, an LED *Z*-loop operator corresponds to a logical-*Z* operator for this qubit, while an *X*-string connecting the interior of the annulus to the exterior corresponds to a logical-*X* operator (Fig. 2a). In this framework, the decay rate *α* of Wilson loops corresponds to a local logical error rate per unit length, and in the correctable phase, LED-based decoding succeeds with high probability as long as the code distance *d* is sufficiently large (Fig. 3c). However, in the uncorrectable phase, such as when *p*_flip_ is above the error correction threshold or when long, open strings condense in a paramagnetic phase, decoding cannot correctly pair anyons, resulting in a high rate of logical errors^23^.

The above results are deeply rooted in the stability of topological order against local perturbations. In contrast, any finite temperature destroys long-range topological order as it leads to freely propagating thermal anyons. In Fig. 3d, we consider the toric code model at finite temperature, with local incoherent errors, and find that the LED loop operators indeed approach zero upon increasing *n*. Interestingly, their expectation values flow non-monotonically, being amplified at small *n* before eventually turning to 0. This occurs because of a competition between two effects: thermal anyons are uncorrectable, so their density accumulates under RG flow; however, local fluctuations are corrected at early layers, which initially amplifies LED loop expectation values. Because loops at different *n* probe correlations at different length-scales, the turning point in these curves can be used to identify the characteristic length-scale of separation between thermal anyons, or equivalently, the system’s temperature.

### Experimental realization in Rydberg atom arrays

**Fig. 4:**
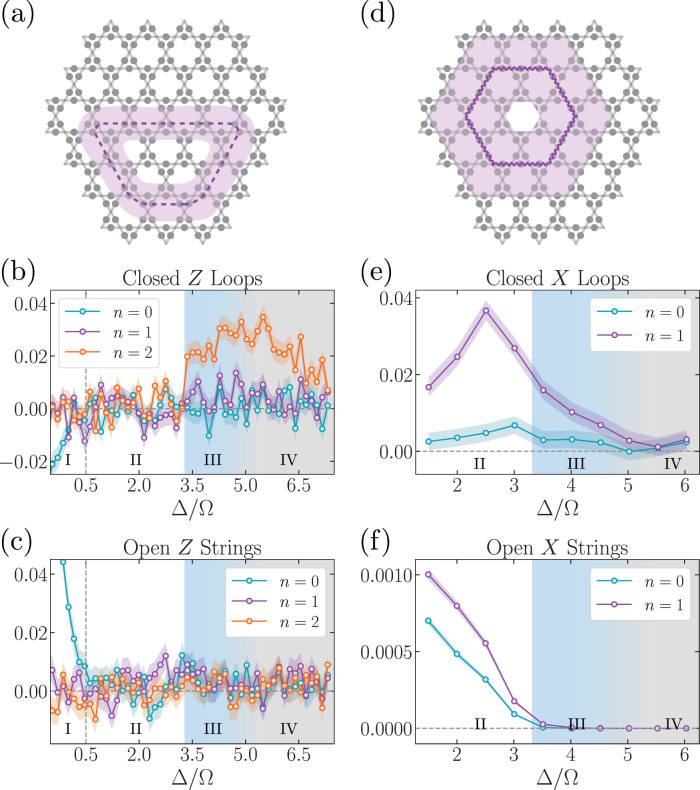
Enhancing experimental detection of ${{\mathbb{Z}}}_{2}$ spin liquid. **a**, **d** In the experiment^28^, 219 qubits are placed on the links of a kagome lattice. Upon applying LED, the *Z* and *X* closed-loop observables are amplified for certain ranges of Δ/Ω. The shaded purple regions show the support of large, decorated Wilson loops after one layer of correction with *n* = 1. **b**, **e** Expectation value of Wilson loops depicted in (**a**, **c**) for different correction layers *n*. Plotted error bars (shaded regions) show expected variation (one standard error) of the mean. The regime in which both types of loops are amplified corresponds nicely to the spin-liquid regime identified in ref. ^28^ (shaded blue region). **c**, **f** The behavior of expectation values of open *Z*- and *X*-strings under LED further confirms our findings, as both types of open strings stay at 0 in the spin-liquid regime. Here, the measured open strings are half of the Wilson loops. By considering the behavior of all types of loops and strings---closed and open, *Z* and *X*---we find that there are four regimes (I-IV), corresponding to four phases: (I) *Z*-paramagnet, (II) *X*-paramagnet, (III) topological spin liquid (blue), and (IV) a phase which is consistent with strong decoherence effects (gray). In our analysis, the progression from Regime (III) to (IV) appears to be smooth.

We now use LED to characterize and provide new insights into the ${{\mathbb{Z}}}_{2}$-topologically ordered states recently realized on a 219-qubit programmable quantum simulator^28^. In the experiment, qubits are encoded in ground states and *n* = 70 Rydberg states of neutral ^87^Rb atoms and placed in an array on the links of a kagome lattice (Fig. 4). This model maps onto a dimer model, where each Rydberg atom can be viewed as a dimer covering the two adjacent vertices of the kagome lattice^29^: the Rydberg blockade interaction between nearby atoms enforces a “dimer constraint” by preventing, with high probability, any vertex from being covered by more than one dimer^30^.

This dimer model is predicted to support a ${{\mathbb{Z}}}_{2}$-topologically ordered state of the resonating valence bond (RVB) type, involving the equally weighted superposition of all dimer coverings^29,31,32^. In this model, *Z*-stabilizers are given by (−1) times the product of single-qubit *Z*-operators on the edges touching a vertex, *X*-stabilizers are given by the product of off-diagonal operators supported on the triangles bordering a hexagon (see Methods), and the RVB state forms a fixed-point state. An *e* (resp., *m*) anyon arises when a *Z* (*X*) stabilizer is violated^33–35^. Here, the (−1) factor for *Z*-stabilizers ensures stabilizer expectation values of +1, because each vertex is touched by exactly one dimer.

In the experiment, a topologically ordered state is prepared by quasi-adiabatically adjusting the detuning Δ and Rabi frequency Ω of a global laser drive^28^. The onset of topological order is observed by studying the expectation values of Wilson loops and open strings^24,28,29,36,37^. A state consistent with ${{\mathbb{Z}}}_{2}$ topological order emerges when using a quasi-adiabatic sweep from initial Δ/Ω < 0 to a final value of Δ/Ω in the range 3.3 ≲ Δ/Ω ≲ 4.5. In practice, several factors make quantitative characterization of such states difficult, as they cause the prepared state to differ from the ideal fixed-point state for the dimer model. In particular, the finite sweep speed and experimental imperfections (e.g., off-resonant scattering, laser phase noise, spontaneous emission events) can modify the experimentally created state. Moreover, the Rydberg interaction Hamiltonian is only an approximation of the parent Hamiltonian of the fixed-point state: for example, the 1/*r*^6^ interaction between Rydberg atoms gives rise to long-range tails in the interaction Hamiltonian. These long-range tails also destabilize the spin-liquid ground state, which could cause a first-order phase transition between regions (II) and (IV) in Fig. 4. Nonetheless, a spin-liquid state can be prepared by using finite ramp speeds, as was done in the experiments^29,38,39^. These factors correspond to both coherent and incoherent perturbations, similar to those considered in our toric code simulations. As a result, while topological order can be discerned at modest length-scales, the expectation values of large, bare Wilson loop observables have nearly vanishing signal for almost all final values of Δ/Ω (Fig. 4b, e).

To circumvent these imperfections, we measure LED loops on the experimentally prepared states. Due to the limited experimental system size, it is not possible to consider loops that strictly satisfy the limit where *ξ* ≪ *d* ≪ *L*, resulting in relatively small expectation values for the LED loop operators. Nonetheless, we clearly observe a range of values of Δ/Ω where both *Z*- and *X*-loops are amplified, which corresponds to the spin-liquid interval identified in ref. ^28^ (blue shaded region in Fig. 4). In particular, some of the largest loops within the system acquire non-zero expectation values in this parameter regime. To further confirm our findings in this intermediate system size setting, we also examine the behavior of open *Z*- and *X*-strings under LED, and we find that there are four regimes (I–IV). Regimes I, II, and III correspond to the *Z*-paramagnet, *X*-paramagnet, and spin-liquid regime, in agreement with the prior interpretation of experimental results^28^. We emphasize that our analysis of Regime III goes beyond that of^28^, showing non-trivial coherence in closed loops at significantly longer length-scales. Furthermore, LED provides novel insights into the nature of Regime IV: because LED does not amplify open or closed string expectation values, our analysis appears to be consistent with a decoherence-dominated disordered phase (see also Supplementary Information). Such a phase is analogous to the disordered part of the mixed-state phase diagram (Fig. 3c), which has a high density of dephasing (*Z*) errors, in contrast to the valence-bond solid (VBS) phase predicted for the ground state^29^.

### Circuit-based LED and generic topological phases

While our current LED analysis uses classical post-processing of *Z*- and *X*-basis experimental snapshots, the most generic LED formulation involves a quantum circuit model following the QCNN framework of ref. ^17^. Here, the entropy associated with both incoherent and coherent fluctuations are systematically removed by introducing ancillary degrees of freedom and applying local unitary transformations, ultimately leaving a purified state supported on fewer degrees of freedom. Notably, this enables the application of LED to a large class of non-abelian topological orders known as string-net models^16,40^. The anyon content of these models is characterized by a *modular tensor category (MTC)*${{{{{{{\mathcal{C}}}}}}}}={{{{{{{\mathcal{Z}}}}}}}}({{{{{{{\mathcal{A}}}}}}}})$, where ${{{{{{{\mathcal{Z}}}}}}}}$ denotes the Drinfeld center^41,42^ of a unitary fusion category ${{{{{{{\mathcal{A}}}}}}}}$^16^. Here, the possible topological charges (i.e., anyon types) are given by the simple objects *α*_0_, *α*_1_, . . . , *α*_*N*−1_ of ${{{{{{{\mathcal{C}}}}}}}}$. It is conjectured that any MTC is uniquely determined by modular *S* and *T* matrices which capture its anyon braiding statistics:34where *d*_*i*_ is the quantum dimension of *α*_*i*_ and ${{{{{{{\mathcal{D}}}}}}}}=\sqrt{{\sum }_{i}{d}_{i}^{2}}$. For example, a key signature of the toric code MTC ${{{{{{{\mathcal{C}}}}}}}}={\mathfrak{D}}({{\mathbb{Z}}}_{2})$ is the − 1 twist product between *e* and *m* anyon loops (*s*_*em*_ = −1).

**Fig. 5:**
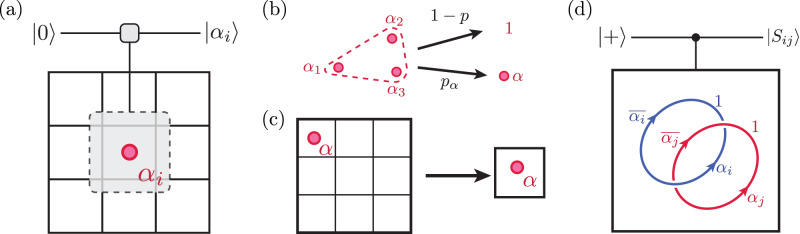
LED for generic string-net models. **a** An ancilla qudit is used to measure the topological charge within each local region ${{{{{{{\mathcal{R}}}}}}}}$: we initialize the ancilla in $\left\vert 0\right\rangle$, apply a local unitary $U=\mathop{\sum }\nolimits_{i,j=0}^{N-1}\left\vert (i+j)\,{{{{{{{\rm{mod}}}}}}}}\,N\right\rangle {\left\langle j\right\vert }_{{{{{{{{\rm{anc}}}}}}}}}\otimes {P}_{i}$, where *P*_*i*_ projects ${{{{{{{\mathcal{R}}}}}}}}$ onto the subspace with topological charge *α*_*i*_, and finally measure the ancilla’s state. **b** Local error correction is performed by inputting the fusion rules of ${{{{{{{\mathcal{C}}}}}}}}$ into a maximum-likelihood patch-based decoder. Given any *l* × *l* patch, one identifies possible groupings of anyons (including groupings to the boundary) that can remove all nontrivial topological charges within the patch. The decoder performs the grouping of highest probability by fusing anyons or dragging them to the boundary of the patch^59^. If ${{{{{{{\mathcal{C}}}}}}}}$ is non-abelian, the vacuum topological charge may only be attained probabilistically with probability 1 − ∑_*α*_*p*_*α*_, or a nontrivial topological charge *α* remains with some probability *p*_*α*_. **c** The system is then coarse-grained by applying a quantum circuit corresponding to a multiscale entanglement renormalization ansatz (MERA) representation of the fixed-point state^43^. **d** At the final layer, *S*- and *T*-matrix elements can be measured by introducing an ancilla qubit in the $\left\vert+\right\rangle$ state and applying controlled-anyon-braiding operations. More details on implementing Steps (**c**) and (**d**) can be found in Methods.

Direct measurements of *s*_*ij*_ and *t*_*ij*_ involve braiding anyons along large loops, and hence are affected by coherent perturbations and incoherent errors. The inability to extract their precise values prevents accurate identification of the topological phase. To circumvent this, we use a hierarchical LED circuit which systematically detects and identifies errors (anyons) at each site by using ancillary qubits, removes them by inputting the fusion rules of ${{{{{{{\mathcal{C}}}}}}}}$ into a maximum-likelihood decoder, and applies an entanglement renormalization circuit to coarse-grain the system^43^. After multiple layers, the *S* and *T* matrices can be measured with much higher accuracy and efficiency (Fig. 5). We note that circuit-based LED is required for the detection and removal of non-abelian anyons. More details on circuit-based LED and generic topological phases can be found in Methods and Supplementary Information.

## Discussion

These results demonstrate that LED constitutes an exceptionally promising approach to enhance the detection and characterization of topological order. Several generalizations and future avenues can be considered. For example, the variational methods of QCNN circuits can enable adaptive measurement procedures, which can recognize a much larger portion of the topological phase. This opens the door towards achieving a necessary and sufficient criterion for topological order using LED, which cannot be done using any fixed linear observable^44^. Moreover, our results indicate that LED is applicable to generic topological orders in higher dimensions, which is challenging to analyze using any currently known techniques. LED can also potentially serve as an order parameter for efficiently characterizing glassy gauge models^45^, through a mapping shown in Methods. In addition, while our present work analyzes a spin-liquid state prepared using a Rydberg-atom quantum simulator, LED is also directly applicable to other platforms such as superconducting qubits^46^ or trapped ions^47^.

Another promising direction is to further study whether the “correctability” of states in our mixed-state phase diagram can be used to characterize topological order in mixed states more generally^11,27,48–50^. In particular, it could be intriguing to further explore the dependence of the correctable regime on the choice of local error correction and/or coarse-graining procedure. Finally, while our approach can be directly applied to any string-net topological order, it could be interesting to consider more general topological phases, fracton phases or gauge theories with continuous gauge groups^35,51^. Such methods can then become indispensable parts of quantum simulation toolboxes for understanding exotic states of entangled quantum matter.

## Methods

### Numerical simulations for the toric code

In this section, we explain how the numerical simulations underlying Figs. 2 and 3 are performed. We begin by constructing a projected entangled pair state (PEPS) representation of the exact toric code ground state^52^. This construction utilizes a parity tensor *P* defined as

5 $$\begin{array}{rc}{P}_{ijkl}&=\left\{\begin{array}{ll}1\quad &{{{{{{{\rm{if\ }}}}}}}}i+j+k+l=0\,{{{{{{\mathrm{mod}}}}}}}\,\,2\\ 0\quad &{{{{{{{\rm{otherwise}}}}}}}}\hfill\end{array}\right.\end{array}$$

where each index *i*, *j*, *k*, *l* ∈ {0, 1} (i.e., the tensor *P* has bond dimension two). Because the toric code is defined with qubits on the links of a square lattice, our PEPS representation of the state has one PEPS tensor with two physical indices per unit cell. Letting *p*, *q* be the physical indices and *ijkl* be the virtual indices, the toric code PEPS tensor *A* is then given by ${A}_{ijkl}^{pq}={\delta }_{i}^{p}{\delta }_{j}^{q}{P}_{ijkl}$. Our perturbed states $\left\vert \psi ({g}_{X},{g}_{Z})\right\rangle$ are constructed from the toric code state by applying imaginary time evolution to each site $L({g}_{X},\, {g}_{Z})={e}^{{g}_{X}X+{g}_{Z}Z}$:

6 $$A{({g}_{X},\, {g}_{Z})}_{ijkl}^{pq}=\mathop{\sum}\limits_{{p}^{{\prime} },{q}^{{\prime} }}L{({g}_{X},\, {g}_{Z})}_{{p}^{{\prime} }}^{p}L{({g}_{X},\, {g}_{Z})}_{{q}^{{\prime} }}^{q}{A}_{ijkl}^{{p}^{{\prime} }{q}^{{\prime} }}.$$

Notice that this operation does not change the PEPS bond dimension, thereby allowing for efficient simulation.

Our goal is to simulate projective *Z*-basis measurements to serve as the “experimental snapshot” input in Fig. 1b. The key ingredient that enables efficient sampling is an algorithm for efficiently computing marginal and conditional probabilities, which can be implemented as follows: We first label every unit cell by its coordinate (*x*, *y*). There are four possible measurement outcomes at each unit cell, and we compute the probability *P*(*σ*_(1, 1)_ = *ab*) that measurement of the first site (*x*, *y*) = (1, 1) yields the outcome *ab* = 00, 01, 10, or 11. Next, we select a sample *ab*_11_ based on this probability distribution, compute the conditional probability distribution on the second site, *P*(*σ*_(2, 1)_ = *ab*∣*σ*_(1, 1)_ = *ab*_11_), and sample the second measurement outcome *ab*_21_. The process then repeats, with each subsequent distribution being conditioned on all prior measurements.

**Fig. 6:**
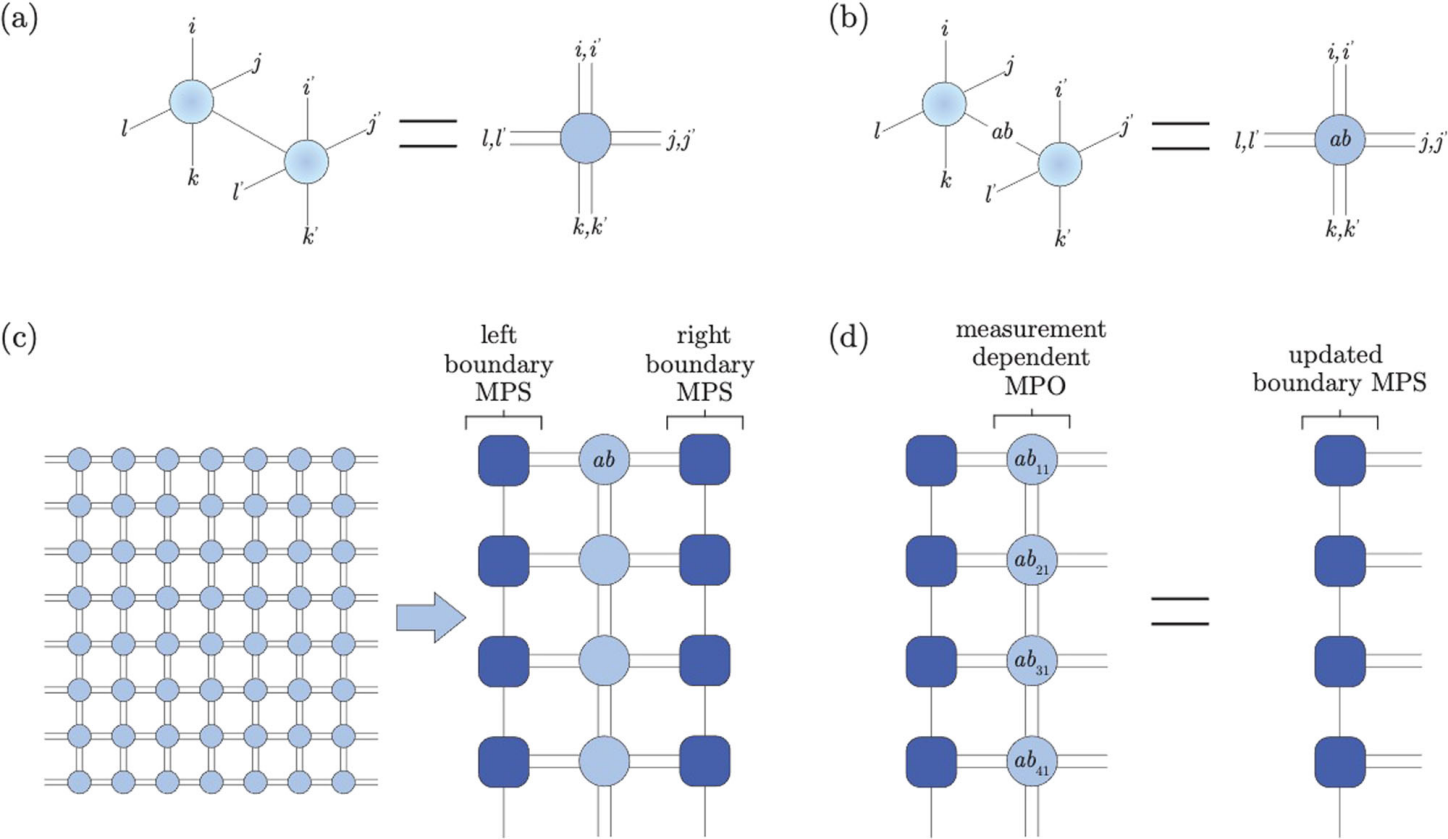
PEPS sampling algorithm. Expectation values are computed with respect to both $\left\vert \psi \right\rangle$ (back) and $\left\langle \psi \right\vert$ (front). **a** Tracing, or averaging over measurement outcomes can be done by contracting the physical indices, and is needed to compute marginal probabilities. **b** To compute the probability of a particular *Z* basis measurement, the physical index is assigned a particular value *ab*. **c** We can efficiently contract a 2D PEPS tensor network on an infinite strip of finite height, by using a left and right boundary MPS (only top four rows shown). The probability distribution for projective measurements on a particular site, e.g., *xy* = 11, can then be computed efficiently. **d** Once an entire column has been sampled, the measurement-dependent MPO can applied to the boundary MPS. Although performing this contraction exactly causes the bond-dimension to grow rapidly, away from phase boundaries, finite bond dimension is sufficient for accurate simulation. See Supplementary Information for more details.

Computing the probabilities requires contracting a 2D tensor network (Fig. 6), which is in general *#P*-hard^53^. In practice, however, the states we encounter have finite correlation length, and the computation becomes remarkably efficient throughout much of the phase diagram^54^. In particular, we work on a strip of finite height *L*_*x*_ and infinite length *L*_*y*_, and introduce boundary matrix product states (MPS) to efficiently capture the effect of the environment—that is, the sites different from the one currently being sampled^55^. Because singular-value decomposition truncation is used at each step to prevent the bond dimension of the boundary MPS from growing exponentially^56^, the method is approximate; however, we only discard singular values < 10^−8^, so truncation errors are insignificant. Details of the boundary conditions and contraction ordering are discussed in the Supplementary Information.

In our simulations, we choose *L*_*x*_ = 300 unit cells and sample 1000 columns, giving us access to very large snapshots with 600,000 qubits. To minimize boundary effects, we compute observables supported on sites at least 30 unit cells away from the boundaries. Near the phase boundaries, the bond dimension (entanglement) of the boundary MPS becomes large due to the large correlation length, which increases the computational demands for sampling (gray data points in Fig. 2g). We numerically confirm this phase boundary with an independent calculation (see Supplementary Information).

### Details on error-correction and coarse-graining procedures

**Fig. 7:**
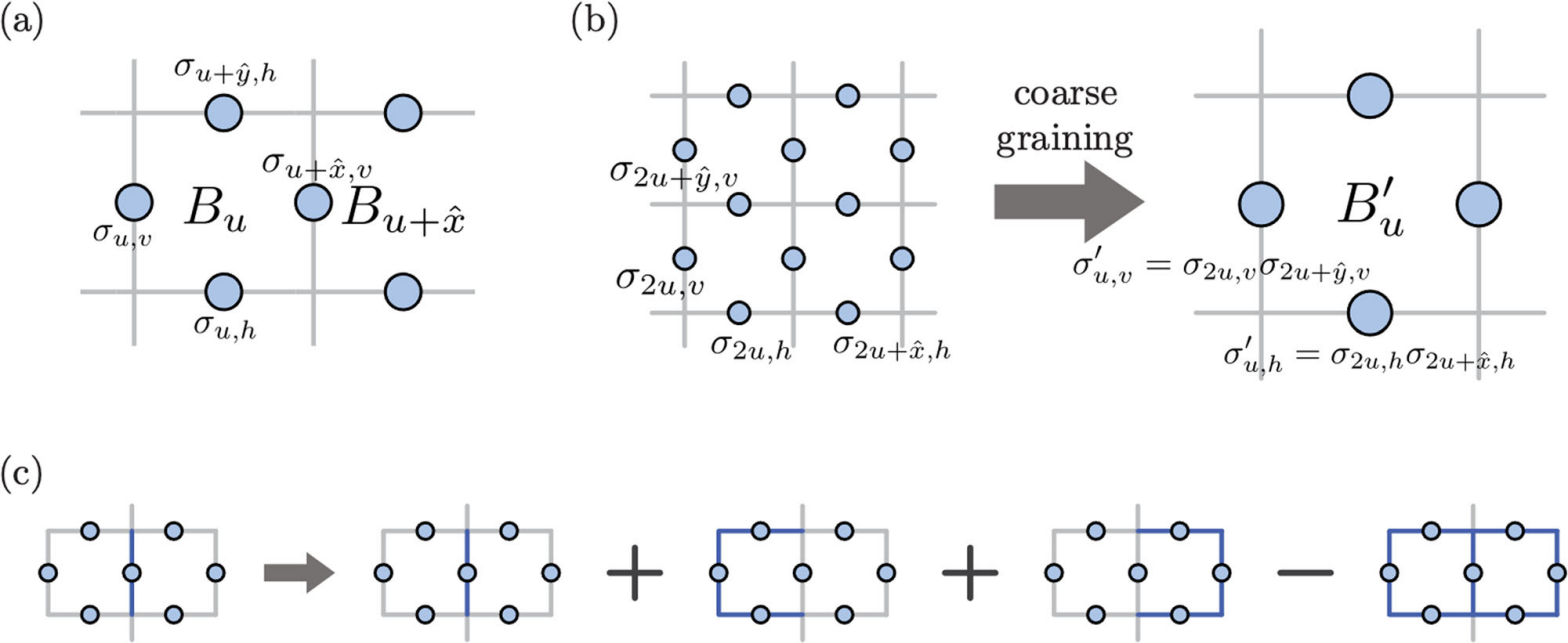
LED coarse-graining and operator transformation. **a** In the toric code model, qubits are located on the links of a square lattice, and the stabilizer associated with any plaquette is given by a product of four single-qubit Pauli-*Z* operators. **b** Coarse-graining maps a *b* × *b* block of plaquettes to a single plaquette whose value is the product of the *b*^2^ plaquettes (here *b* = 2). Microscopically, coarse-grained qubits ${\sigma }^{{\prime} }$ are products of *b* lower-level qubits *σ*. Coarse-grained stabilizers ${B}_{u}^{{\prime} }$ are therefore equivalent to a product of *b* × *b* stabilizers at the lower level. **c** Pairing correction flips a qubit conditioned on the state of its two neighboring stabilizers. This is equivalent to an operator transformation where the qubit is decorated by products of closed loops.

Here, we explain the details of the LED decoding and coarse-graining procedures and demonstrate how bare Wilson loops become decorated under the LED protocol. Without loss of generality, we consider *Z*-basis measurements, from which we can calculate plaquette stabilizers *B*_*u*_. Here, each plaquette is labeled by the 2D coordinate of its unit cell *u* = (*x*, *y*). Since there are two qubits per unit cell, each qubit carries a coordinate and a link label *v* or *h*, depending on whether its corresponding edge in the square lattice is vertical or horizontal, respectively. Finally, the projective measurement outcomes are denoted by *σ* ∈ {+1, −1} (Fig. 7).

To illustrate local error correction, we consider the “pairing decoder,” which flips a qubit if and only if its two neighboring plaquettes are simultaneously occupied. Importantly, to preserve locality, we first compute all stabilizer values and then flip qubits based on these values. The decision of whether to flip any qubit then depends only on its value, and the values of the six adjacent qubits with which it shares a plaquette. Equivalently, this error correction procedure corresponds to an operator transformation

7 $${\sigma }_{u+\hat{x},v}\to {\sigma }_{u+\hat{x},v}\left(1+{B}_{u}+{B}_{u+\hat{x}}-{B}_{u}{B}_{u+\hat{x}}\right)/2$$

8 $${\sigma }_{u+\hat{y},h}\to {\sigma }_{u+\hat{y},v}\left(1+{B}_{u}+{B}_{u+\hat{y}}-{B}_{u}{B}_{u+\hat{y}}\right)/2$$

To ensure all local errors are removed after a finite number of LED steps, we also pair anyons, which occupy two plaquettes separated by a diagonal, such as *B*_*u*_ and ${B}_{u+\hat{x}+\hat{y}}$. The locality of the decoder ensures that the support of any local operator only grows by a finite amount with each step. Subsequently, the coarse-graining procedure replaces each *b* × *b* block of plaquettes with a single plaquette whose value is the product of *b*^2^ plaquettes; microscopically, this can be done by defining new qubits as a product of *b* corresponding qubits in the original lattice. The combination of a local pairing step and a coarse-graining step forms a layer of real-space RG; with each additional layer, one can correct errors of higher and higher weight.

The bare Wilson loops measured in the final state are equivalent to decorated loop operators acting on the original state. These decorated operators can be efficiently computed from projective measurement data, since their eigenstates are product states in the *Z* and *X* bases, respectively. Furthermore, in the operator transformation picture, any loop or string of length *L* maps onto a linear combination of exponentially many (2^*O*(*L*)^) loops or strings, respectively. Thus, while the operator transformation picture is helpful for conceptual reasons, it is computationally much easier to use the original picture of error-correction and coarse-graining.

A few remarks are in order. First, one important property of LED is that it preserves commutation relations: consider two anti-commuting *X* and *Z* strings that intersect at a single point, far from the strings’ endpoints. Upon applying LED, the resulting decorated strings still anti-commute. This is because the correction is computed only using stabilizers, so it decorates *Z*-operators by a linear combination of closed *Z*-loops, and similarly for *X*. Moreover, other local decoding algorithms, such as cellular automata and RG decoders, can also be used to generate different LED operators^57^. In the following section, we describe a flexible, “patch-based” local decoder for the toric code, which allows LED to classify a wider range of states as topological.

### Patch-based decoder

The patch-based decoder with variable correction distance *d* is based on a local minimum-weight perfect matching (MWPM) procedure. In the first decoding step, a local MWPM decoder is convolved with all *l* by *l* square regions of the toric code, where *l* ~ *d*; for each region, MWPM takes as input the location of the enclosed anyons. Because both *e* and *m* anyons can freely move into and out of the region, this is analogous to decoding a surface code with open boundaries. Therefore, MWPM pairs any given anyon either with another anyon or with the boundary.

**Fig. 8:**
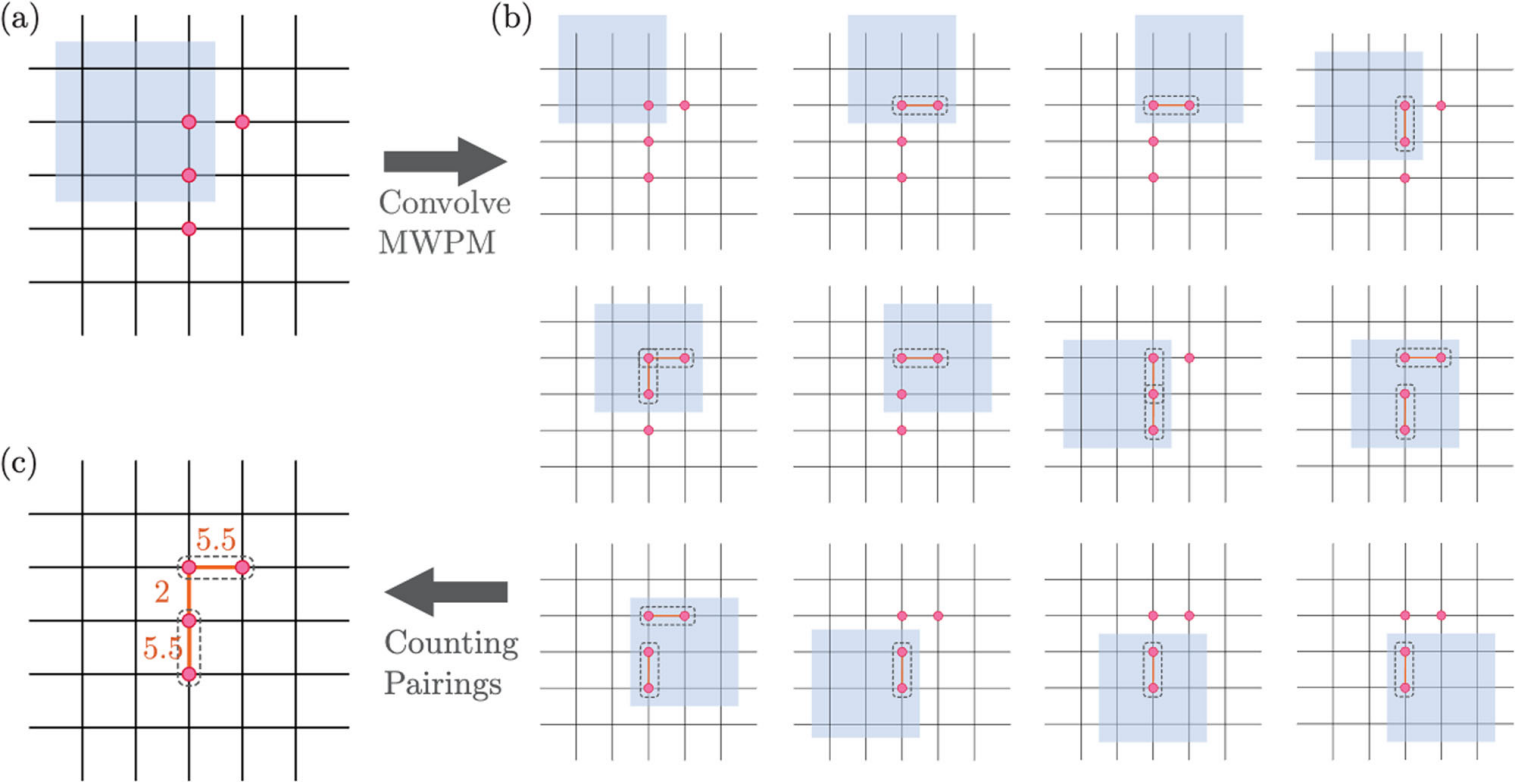
MWPM-based patch decoder. **a** Example of an error chain which creates four *e*-anyons. **b** The decoding algorithm performs correction using only local information by splitting the large system into smaller overlapping regions, within each of which the MWPM algorithm is used to find the lowest-weight pairing of anyons. These local regions have open boundaries, hence MWPM can also pair anyons to the boundaries if this is of lower weight. In practice, a slight boundary bias is added to break ties in favor of boundary pairing. **c** The final step requires locally combining the pairing outputs to determine the final pairing. In particular, we count the number of times each site *p* is paired to sites *q* > *p*. In the diagram, two equal-weight pairings contribute 0.5 each, though we randomly break the tie in practice. Then, the algorithm pairs *p* with the *q* that appears most often. In this example diagram, we connect two pairs that have weight = 5.5, and do not form the weight = 2 pairing. We see in the simple four-anyon case depicted above, the procedure correctly recovers the pairing with windows of size *l* = 3. In general, this patch-based decoder can correct errors up to distance *d* = ⌊*l*/2⌋; moreover, the distance by which it spreads information and the thickness of any associated LED operators are both proportional to *l*.

The second step aggregates MWPM pairings. Since the square regions can overlap, a pair may appear more than once. As such, after choosing a natural indexing of the plaquettes, we create a list of all MWPM pairings between two plaquettes (*p*, *q*) with *p* < *q*; pairings with the boundary are not included (Fig. 8). For each plaquette *p* containing an anyon, the patch-based decoder then performs the pairing (*p*, *q*) which occurs most often. This procedure naturally favors pairings that flip fewer qubits, because shorter-range pairings can be included in more local patches.

A critical property of this decoder is that it preserves locality. In the first step, MWPM only uses information from local *l* by *l* patches, while the distance between partner plaquettes in the second step is always less than *l*. Aggregation can thus be performed using only the results from a small number of overlapping local patches.

### Decoder details for the ruby lattice spin liquid

We now explain the decoding procedure for a dimer model where qubits lie on the vertices of the ruby lattice, or equivalently, on the links of a kagome lattice. This dimer model supports a ${{\mathbb{Z}}}_{2}$ spin-liquid phase, whose fixed-point is a resonating valence-bond (RVB) state^29^. This state is in the same universality class as the toric code, as it supports *e* and *m* anyons with similar string operators.

**Fig. 9:**
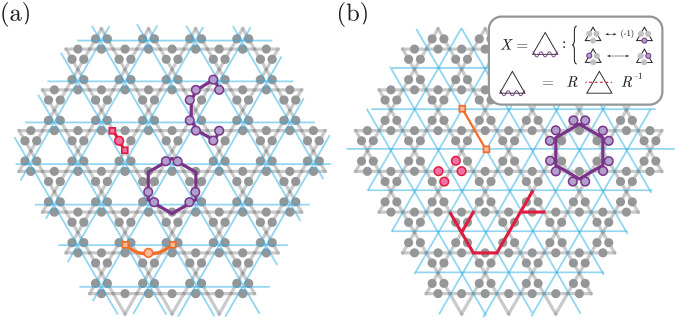
Decoding for the ruby lattice spin liquid realized in ref. ^28^. **a** For *Z*-loops, two layers of LED can be performed. In both layers, we use the pairing decoder, which flips a qubit (e.g., red or orange circle) if and only if both neighboring stabilizers (e.g., red or orange squares) are equal to −1. Stabilizers in the first layer (e.g., red squares) are given by (−1)∏_*i*∈*v*_*Z*_*i*_ for each vertex *v* of the kagome lattice. The coarse-graining procedure after the first decoding step maps three stabilizers to a single stabilizer (e.g., orange square) in the coarse-grained lattice (blue lines), whose value is determined by the product of the qubits along a loop enclosing a triangle (e.g., purple closed loop). The open strings considered in the main text start and end at hexagons (e.g., purple open string). **b** To measure *X*-loops, a basis rotation is first performed within each triangle of the kagome lattice, so that the *X*-string operators become diagonal in the measurement basis (inset and refs. ^28, 29^). Each configuration is then mapped to a triangular lattice (blue lines), where each edge of the triangular lattice is determined by the product of four qubits in the original lattice (e.g., red circles); moreover, the *X* stabilizers of the dimer model become vertex stabilizers in the triangular lattice (e.g., purple hexagons). As before, the pairing decoder flips qubits (orange edges) conditioned on the values of stabilizers (e.g., orange squares). Open strings on the triangular lattice also map to open strings in the kagome lattice (e.g., red string), although the resulting strings are slightly different from the ones measured in refs. ^28, 29^.

We first describe the decoding procedure for *e* anyons, which correspond to vertices with an even number of adjacent dimers. (We note that this is an odd *Z*_2_ spin liquid, and the trivial empty state corresponds to maximal occupation of *e* anyon states.) In the first correction step, we apply the pairing decoder between adjacent vertices. We then coarse-grain the kagome lattice to a triangular lattice by grouping vertices within each upward-pointing triangle. This transforms vertex stabilizers in the kagome lattice to vertex stabilizers in the triangular lattice (Fig. 9a). The pairing decoder is then applied between adjacent triangles in the second correction step. In the main text, we study the flow from uncorrected loops to vertex-paired and triangle-paired loops, which are denoted as as layers 0, 1, and 2, respectively.

We next consider the *m* anyons, which are associated with hexagonal plaquettes. A rotation is first performed within each triangle, such that the string operators associated with *m* anyons become diagonal in the measurement basis. This allows us to map each configuration onto a triangular lattice, whose vertices are located at the center of each hexagon in the kagome lattice; this mapping transforms *X*-stabilizers of the dimer model into vertex *Z*-stabilizers in the triangular lattice (Fig. 9b). Due to the small experimental system size, we can only perform one layer of correction, and we use the pairing decoder on the triangular lattice. We note that open strings on the triangular lattice map onto open strings on the ruby lattice, although the resultant strings are slightly different from the ones measured in refs. ^28, 29^.

### Quantum circuit formulation of LED

As discussed in the main text, the most general formulation of LED uses a hierarchical quantum circuit like the QCNN circuit introduced in ref. ^17^. The structure of such a circuit is illustrated in Supplementary Fig. S11 in a one-dimensional example for simplicity of illustration, but can be easily generalized to the two-dimensional cases considered in LED.

In this framework, stabilizer measurements are performed at each layer using quantum circuits to preserve the coherence of qubits in the system, in the same fashion as for surface-code quantum computation^58^. When the lattice is coarse-grained as in Figs. 1, 7, a fraction of the system’s qubits are measured, and local operations are applied to each remaining qubit based on nearby stabilizer measurement values, to correct for local errors. One example circuit construction of LED stabilizer measurement, decoding, and coarse-graining for recognizing the toric code phase is presented in the Supplementary Information. For more general string-net models, ancillas can be used to detect the presence of anyons, and the decoding steps perform anyon transport and fusion via procedures described in ref. ^59^; meanwhile, the coarse-graining circuit is constructed as the inverse circuit of a multiscale entanglement renormalization ansatz (MERA) representation of the fixed-point state (Fig. 5)^43^. In addition, a layer of variational unitary operations is placed in front of each anyon-detection step.

These variational unitaries can be tuned to optimize the LED order parameter values, especially in the presence of (quasi-)local rotations of qubits on top of a known fixed-point state. For example, if every qubit in a perfectly prepared toric code state underwent a Haar-random, single-qubit operation, both the bare and snapshot-based LED Wilson loop operators will be exponentially small. However, the layer of variational unitaries in front of the first local decoding step enables one to un-do these single-qubit operations and again achieve a high LED signal. In particular, one uses here an adaptive procedure, whereby a hybrid quantum-classical feedback loop is used to tune each unitary to optimize LED loop values. More generally, variational unitaries in front of subsequent local decoding steps *l* allow us to compensate for local operations acting on multiple qubits of the system. This is a major step towards achieving a necessary and sufficient criterion for topological order, which is not possible using a single, fixed observable such as a bare Wilson loop operator^44^. Moreover, due to the special hierarchical structure of QCNN and LED circuits, the optimization of the variational unitaries can be done efficiently without encountering the so-called “barren plateau” challenges of variational quantum circuits^60,61^.

Finally, one other advantage of circuit-based LED is that it enables the simultaneous measurement of loop operators in multiple bases in each experimental repetition. This allows us to capture anyonic braiding statistics, which is critical to the application of LED to non-abelian phases. In particular, the final measurement of *s*_*ij*_ (Fig. 5d) can be performed by initializing an ancilla qubit in the state $\left\vert+\right\rangle=\frac{1}{\sqrt{2}}(\left\vert 0\right\rangle+\left\vert 1\right\rangle )$, and applying a controlled operation which, conditioned on the ancilla being in $\left\vert 1\right\rangle$, creates anyon pairs ${\alpha }_{i},\overline{{\alpha }_{i}}$ and ${\alpha }_{j},\overline{{\alpha }_{j}}$, braids $\overline{{\alpha }_{i}}$ around *α*_*j*_, and fuses the pairs ${\alpha }_{i},\overline{{\alpha }_{i}}$ and ${\alpha }_{j},\overline{{\alpha }_{j}}$. *s*_*ij*_ is then measured in two steps: First, the magnitude ∣*s*_*ij*_∣^2^ is equal to the probability of ${\alpha }_{j},\overline{{\alpha }_{j}}$ fusing to vacuum when the ancilla is in $\left\vert 1\right\rangle$; this probability can be obtained by measuring local energy densities (e.g., by performing stabilizer measurements). Then, when ∣*s*_*ij*_∣^2^ > 0, we post-select on both ${\alpha }_{i},\overline{{\alpha }_{i}}$ and ${\alpha }_{j},\overline{{\alpha }_{j}}$ fusing to vacuum and measure the ancilla’s final state

9 $$\vert {S}_{ij}\rangle \propto \left\vert 0\right\rangle+{s}_{ij}\left\vert 1\right\rangle$$

in an appropriate basis to obtain the phase of *s*_*ij*_.

For topological phases described by Abelian quantum double models^16^, the quantum circuit and snapshot-based versions of LED can be combined by measuring all qubits in a fixed basis after some chosen depth *d*, and performing snapshot-based LED using the resulting stabilizer measurement values (see Supplementary Information). The choice of *d* is then determined by a tradeoff between the quantum circuit depth/fidelity and the generality of local rotations, which can be compensated for.

### Topological order witness

Here, we show that LED provides a topological order witness—that is, it does not misclassify any trivial product state as topological. For simplicity, we study the case of ${{\mathbb{Z}}}_{2}$ topological order on a surface with trivial topology, where the fixed-point state is the unique ground state $\left\vert {\psi }_{{{{{{{{\rm{TC}}}}}}}}}\right\rangle$ of *H*_TC_. We begin by considering the ideal case where LED operators go to one.

#### Theorem 1

Let *ρ* be an arbitrary input state defined on a surface with trivial topology. Then, after performing LED with correction distance *d*, assume the resultant state *ρ*_*f*_ has, as a subsystem, qubits living on the links of a square lattice, as in the toric code. Then, if the stabilizer expectation values $\left\langle \frac{1+{A}_{v}}{2}\right\rangle=\left\langle \frac{1+{B}_{p}}{2}\right\rangle=1$ at every vertex *v* and plaquette *p* of the subsystem, then, the input state *ρ* is topologically ordered, in the sense that it is connected to an output state of the form ${\rho }_{f}=\left\vert {\psi }_{{{{{{{{\rm{TC}}}}}}}}}\right\rangle \left\langle {\psi }_{{{{{{{{\rm{TC}}}}}}}}}\right\vert \otimes {\alpha }_{{{{{{{{\rm{anc}}}}}}}}}$ by generalized local unitary (gLU) transformation of depth *O*(*d*).

The key to the proof is a unitary implementation of LED by introducing product state ancillas and performing local unitary gates to perform stabilizer measurement and correction (see Supplementary Information for details). These operations, which cannot change the long-range entanglement structure of the state, are known as gLU transformations^18^, and preserve phase boundaries. Thus, if we further assume the output ancillas *α*_anc_ are in a trivial state, Theorem 1 guarantees the input state is in the toric code phase. However, we do not certify this condition holds, which is in general more difficult: measurements in multiple bases are needed to uniquely determine *α*_anc_. Instead, LED certifies that the toric code state can be *distilled* from the input state by gLU transformations. Because long-range entanglement cannot be created from a trivial state by gLU transformations^18^, Theorem 1 implies that LED operators flowing to unity forms a sufficient condition for topological order, or equivalently, a topological order witness (see also ref. ^7^).

While the above argument works well in theory, any practical system cannot measure LED observables equal to one with infinite precision. Indeed, even infinitesimal local perturbations to the toric code ground state, such as ${e}^{-i\epsilon H}\left\vert {\psi }_{{{{{{{{\rm{TC}}}}}}}}}\right\rangle$ for arbitrarily small *ϵ* and some local Hamiltonian *H*, can create error strings larger than the correction length *d*. This causes LED loop expectation values to decay exponentially, even in the topological phase. To show that LED still provides a topological order witness in the presence of local perturbations, finite measurement errors, and finite system size, we show the following Theorem:

#### Theorem 2

Consider an arbitrary input state *ρ* and LED with correction distance *d*, as in Theorem 1. Suppose the corresponding subsystem of *ρ*_*f*_ has stabilizer expectation values $\left\langle \frac{1+{A}_{v}}{2}\right\rangle > 1-\epsilon$, $\left\langle \frac{1+{B}_{p}}{2}\right\rangle > 1-\epsilon$ at every vertex *v* and plaquette *p*. Then, the input state *ρ* exhibits topological ordering at least up to a length-scale $O({{{{{{{\mathscr{L}}}}}}}}-d)$; that is, no purification of *ρ* can be prepared using a local quantum circuit of depth less than $O({{{{{{{\mathscr{L}}}}}}}}-d)$, where ${{{{{{{\mathscr{L}}}}}}}} \sim 1/\sqrt{\epsilon }$.

Our proof of Theorem 2 hinges on the following two Lemmas, proved in the supplement.

#### Lemma 3

Given an output state *ρ*_*f*_ satisfying the conditions of Theorem 2, and a simply connected $({{{{{{{\mathscr{L}}}}}}}}-2)\times ({{{{{{{\mathscr{L}}}}}}}}-2)$ square region *R* on the system part, the reduced density matrix ${\rho }_{d}={{{{{{{{\rm{Tr}}}}}}}}}_{{R}^{c}}[{\rho }_{f}]$ is indistinguishable from the toric code reduced density matrix ${\sigma }_{{{{{{{{\rm{TC}}}}}}}}}={{{{{{{{\rm{Tr}}}}}}}}}_{{R}^{c}}[\left\vert {\psi }_{{{{{{{{\rm{TC}}}}}}}}}\right\rangle \left\langle {\psi }_{{{{{{{{\rm{TC}}}}}}}}}\right\vert ]$ defined on the same region, up to the bound $| | {\rho }_{d}-{\sigma }_{{{{{{{{\rm{TC}}}}}}}}}| | \le \max \left(\sqrt{\epsilon },2{{{{{{{{\mathscr{L}}}}}}}}}^{2}\epsilon \right)$.

#### Lemma 4

Consider an input state *ρ* and an LED procedure satisfying the conditions of Theorem 2. Then the final state *ρ*_*f*_ after LED cannot be prepared using a local quantum circuit with depth less than $O({{{{{{{\mathscr{L}}}}}}}}) \sim O(1/\sqrt{\epsilon })$.

Upon combining the result of Lemma 4 with the fact that our LED procedure corresponds to a local quantum circuit with depth *O*(*d*), we find that the original input state $\left\vert \psi \right\rangle$ cannot be prepared using a quantum circuit of depth smaller than $O({{{{{{{\mathscr{L}}}}}}}}-d)$—which is precisely the statement of Theorem 2. So, if we measure loops of length *L* ≫ *d* to be 1 − *ϵ*, this shows that LED provides a topological order witness up to length-scales of $O(L/\sqrt{\epsilon })$.

**Fig. 10:**
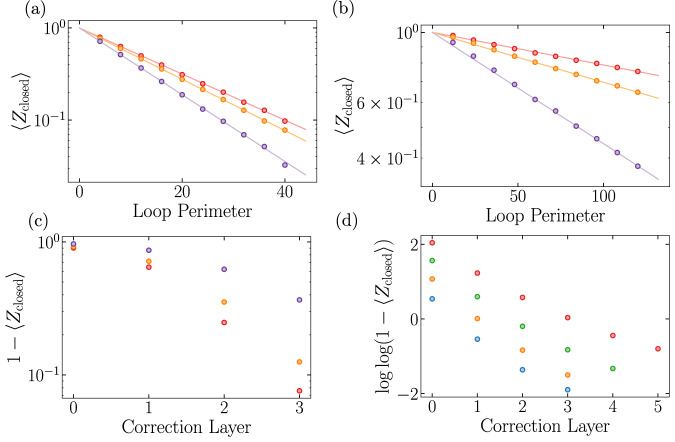
Perimeter-law decay of Wilson loops is clearly visible at various points in the topological phase—(orange) *g*_*Z*_ = 0, *g*_*X*_ = 0.18, *p*_flip_ = 0, (red) *g*_*Z*_ = 0.18, *g*_*X*_ = 0.18, *p*_flip_ = 0, (purple) *g*_*Z*_ = 0.10, *g*_*X*_ = 0.18, *p*_flip_ = 0.03. This is observed for both (**a**) uncorrected loops and (**b**) *d* = 6 corrected loops under two layers of *d* = 3 MWPM patch decoding. **c** LED Wilson loops appear to approach one faster than exponential in *n*. **d** In a model with only incoherent errors (*p*_flip_ = 0.02 (blue), 0.03 (orange), 0.04 (green), 0.05 (red)), we can study the effect of even more layers, where we see hints that the decay is doubly exponential in *n*, or exponential in *d* ~ 2^*n*^.

We now discuss how these theoretical results are reflected in our numerical simulations. First, when fluctuations are local, the probability of having an error string of length *ℓ* decays exponentially with *ℓ*, and the exponent is determined by the characteristic length-scale *ξ* of fluctuations. In these systems, we expect the error rate after an optimal LED procedure with correction distance *d* to be given by *ϵ*(*d*) ∝ *e*^−*d*/*ξ*^, so correction distance $d={{\Omega }}(\xi \log {{{{{{{\mathscr{L}}}}}}}})$ is sufficient to certify topological order up to length-scale ${{{{{{{\mathscr{L}}}}}}}}$. Second, when LED uses the hierarchical, anyon-pairing decoder, the anyon density is observed to decrease faster than exponentially in the number *n* of LED steps (Fig. 10). In this case, both the measured stabilizer size and the correction distance *d* grow exponentially with *n*, which implies that the certification length-scale ${{{{{{{\mathscr{L}}}}}}}}$ grows at least exponentially with *n* as well. Third, our argument does not certify topological order to any length-scale when *L* < *d*; this is because the support of such an LED operator no longer has an interior, potentially giving rise to signal even in the trivial phase. Indeed, this is reflected in our numerics as well (Supplementary Information, Fig. S12).

### Connection to topological entanglement negativity

The entanglement negativity of a mixed state *ρ*_*S*_ is defined as ${S}_{N}(\rho )=\log | | \rho | {| }_{1}=\log (\sum {\lambda }_{i})$, where *λ*_*i*_ are the eigenvalues of *ρ*. Prior works have shown, via a combination of analytical arguments and numerical results, that in a topological phase, *S*_*N*_ obeys an area-law with a constant correction, i.e. *S*_*N*_ = *αL* − *γ*. Further, recent results have also shown that the topological term *γ* vanishes at finite-temperature^62^, or for high incoherent error rates^63^. Thus, the negativity appears to capture important features of mixed state topological order.

The unitary circuit construction of LED also enables us to connect a positive classification under LED, to the topological entanglement negativity of the input state. In particular, theorem 1 implies that states classified as topological are connected to an output state ${\rho }_{f}=\left\vert {\psi }_{{{{{{{{\rm{TC}}}}}}}}}\right\rangle \left\langle {\psi }_{{{{{{{{\rm{TC}}}}}}}}}\right\vert \otimes {\alpha }_{{{{{{{{\rm{anc}}}}}}}}}$ via local unitary circuits. If we further assume the ancillas contain no long-range order (see SM for rigorous definition), then since $\left\vert {\psi }_{{{{{{{{\rm{TC}}}}}}}}}\right\rangle$ is topologically ordered, the output state indeed has a topological correction in the entanglement negativity. It is further believed that *γ* is a topological invariant, i.e., it should remain invariant under local unitary circuits. As such, this should be sufficient to certify the input state *ρ*_*S*_ has topological order.

We show this in the SM, for the special case where the LED circuit is composed of Clifford gates, by extending the stabilizer formalism introduced in ref. ^62^. Interestingly, there, the topological correction to *S*_*N*_ comes from the presence of decorated Wilson loops operators with non-trivial twist product in the input state *ρ*_*S*_ (see also proof of Lemma 4). Thus we conjecture a connection to topological entanglement negativity holds for LED Wilson loops more generally.

### Supplementary information

Supplementary Information

Peer Review File

## Data Availability

The data that support the plots within this paper and other findings of this study are available at https://osf.io/k8up2/. We note that more extensive data from the Rydberg-atom spin liquid experiment of ref. ^28^ are available at https://dataverse.harvard.edu/dataset.xhtml?persistentId=doi:10.7910/DVN/BDCTRX.

## Code availability

The data that support the plots within this paper and other findings of this study are available at https://osf.io/k8up2/. We note that more extensive data from the Rydberg-atom spin liquid experiment of ref. ^28^ are available at https://dataverse.harvard.edu/dataset.xhtml?persistentId=doi:10.7910/DVN/BDCTRX.
