# Supplementary material for: Enhancing detection of topological order by local error correction
Source: Nat Commun. 2024 Feb 20;15:1527. doi: 10.1038/s41467-024-45584-6 (PMC10879205; doi:10.1038/s41467-024-45584-6)
Supplement: Supplementary file 1 — Supplementary Information [file 41467_2024_45584_MOESM1_ESM.pdf]

SUPPLEMENTARY INFORMATION

Contents

Supplementary Methods 1
PEPS Sampling Contraction Details 1
Calculation of Phase Diagram 1
Sample Complexity 2
Decoder Dependence 3

Supplementary Discussion 3
LED Circuit for $\mathbb{Z}_2$ Toric Code 3
LED for Abelian Quantum Double Models 5
Arbitrary Local Perturbations 6
Proof of Theorem 1 6
Proof of Lemmas 3 and 4 7
Connection Between LED and Entanglement Negativity 9
Valence-Bond Solid Phase 12
Detecting Gauge-Glass Phases and More 13
Annulus Decoders 13

Supplementary Notes 14
Definition and Properties of Fixed-Point States 14
Background on Generic Topological Phases 14

References 15

SUPPLEMENTARY METHODS

PEPS Sampling Contraction Details

We start by computing the left boundary MPS of an infinite strip. For sites with $y \leq 0$, we average over measurement outcomes, and hence the doubled PEPS tensor for each site, which contains both the bra and the ket tensors, is $T^{i',j',k',l'}_{i,j,k,l} = \sum_{pq}(A^*)^{pq}_{i',j',k',l'}A^{pq}_{i,j,k,l}$. The boundary conditions we choose are $|+\rangle_i |+\rangle_{i'}$ at the lower $x = 1$ boundary and $\delta_{i,i'}$ at the $x = L_x$ boundary. Contracting the doubled tensor along an entire column $x = 1, ..., L_x$ results in a matrix product operator (MPO) T_y acting on the boundary MPS. Then, we can compute the effect of an infinite environment by repeatedly applying T_y to some initial boundary MPS until it converges. The right boundary MPS can be similarly computed, by exchanging the input and output directions of the MPO. Note that at each application of T_y, we use singular-value decomposition truncation to prevent the bond dimension of the boundary MPS from growing too rapidly, rendering the method approximate. However, only singular values smaller than $< 10^{-8}$ are discarded, so truncation errors should be insignificant. We refer to the resulting tensors as the left and right fixed-point of T_y.

The algorithm continues by using the left and right boundary MPS to sample a column of sites. Computing the marginal probability $P(ab)$ on the first site requires contracting a 1D tensor network (see Figure **??**), where the doubled tensor at $x, y = 1, 1$ is replaced by a measurement-dependent one $T(ab)^{i',j',k',l'}_{i,j,k,l} = (A^*)^{ab}_{i',j',k',l'}A^{ab}_{i,j,k,l}$, while the doubled tensor at sites $x > 1$ remain measurement-independent. Note that the tensor network outputs an unnormalized probability distribution; however, since there are only four states per site, the normalization can be computed with little overhead. After drawing a sample ab_{11} for the first site, we replace the doubled tensor at $(x, y) = (1, 1)$ by $T(ab_{11})$, and then compute the distribution of the second site. The process is repeated until the entire column is sampled. To minimize repeat 1D contractions, upper and lower environment tensors can be stored and updated during the sweep $x = 1, ..., L_x$.

Next, the history of samples along the column are used to construct a measurement-dependent MPO $T_y(ab_1, ab_2, ..., ab_{L_x})$, where the doubled tensor at each site is replaced by a measurement-dependent one. Then, the left boundary MPS can be updated by contraction with $T_y(\{ab_i\})$, and the process repeated for the second column. Thus, the left boundary MPS keeps track of the effect of past measurements on future measurements, as the algorithm sweeps from left to right. Meanwhile, the right boundary MPS remains unchanged, as it is modelling a static, infinite environment.

Calculation of Phase Diagram

To compute the phase diagram, we use the PEPS tensors to construct a transfer matrix with periodic boundary conditions, on small cylinders with circumference L_y measured in unit cells. The largest few eigenvalues of the transfer matrix can be efficiently computed using Krylov algorithms in this regime, and the degeneracy of the largest eigenvalue serves as an alternative signature of the topological transition [1, 2]. In particular, the local $\mathbb{Z}_2$ gauge symmetry of the PEPS tensor becomes a $\mathbb{Z}_2 \times \mathbb{Z}_2$ symmetry of the doubled tensor (bra and ket). Hence the topological, e-condensate (Z-paramagnet), and m-condensate (X-paramagnet) correspond to three distinct symmetry broken phases from the point of view of the virtual legs, with degeneracy two, one, and four respectively.

As such, along the transition from topological to e-condensate, which occurs for g_X small and $g_Z \approx 0.2-0.3$, the relevant ratio is between the first and second eigenvalues, $\Delta_{12}(g_Z, g_X) = \log(\lambda_1/\lambda_2)$. In contrast, for the

transition from topological to m-paramagnet, the relevant gap is between the first and third eigenvalues $\Delta_{13}(g_Z, g_X)$. Furthermore, the model is self-dual, so the wavefunction at $|\psi(g_z, g_x)\rangle$ is equivalent to the wavefunction at $|\psi(g_x, g_z)\rangle$ by a basis rotation and spatial translation. We will use this duality to compute the phase boundary in a way which minimizes finite-size effects, by computing the two gaps at their dual points (Fig S1a). Therefore, we introduce two parameters g_0, g_1, and let g_1 be the parameter which changes across the transition. The relevant parameter is therefore $\Delta_{12}(g_1, g_0)$ for the topological to e-condensate transition, and $\Delta_{13}(g_0, g_1)$ for the topological to m-condensate transition.

As Δ_{12} grows with increasing g_1, while Δ_{13} reduces with increasing g_1, these two ratios will eventually cross. Indeed, in the limit $L_y \to \infty$, the crossing point should exactly correspond to the phase boundary. In general, finite L_y may shift the boundary. Empirically, we see that at $g_0 = 0$, the solvable point, there are essentially no finite size effects, and the agreement with the analytical value $g_c = 0.2203434$ is almost exact (Fig. S1ac). For larger g_0, the dependence on L_y appears minimal until around $g_0 = 0.01$: indeed, even in this case, $L_y = 4$ only overestimates the phase boundary compared to $L_y = 6$ by a few percent (Fig. S1d). As such, we use $L_y = 4$ and compute the phase boundary by identifying points where the difference $\Delta_{12} - \Delta_{13}$ is close to zero (Fig S1b).

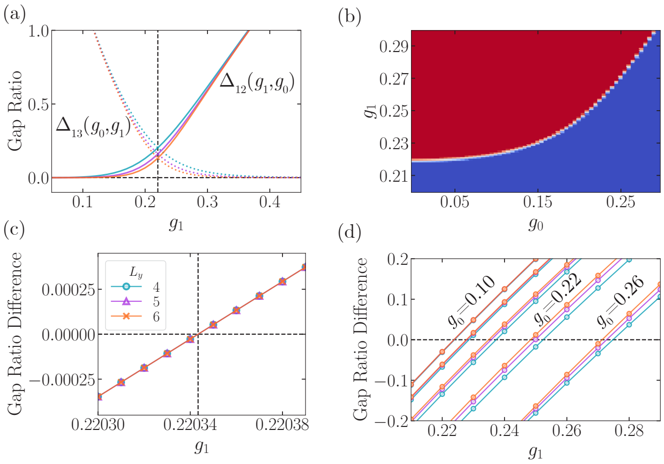

FIG. S1: (a) For a given point (g_0, g_1) on the phase diagram, the phase transition from topological to e-condensate is signaled by the transfer matrix gap ratio $\Delta_{12}(g_0, g_1)$, while the transition from topological to m-condensate is signaled by $\Delta_{13}(g_1, g_0)$, where both quantities are evaluated at the self-dual point with $g_z \leftrightarrow g_x$ switched. When these two gaps are equal, the system must be at the critical point, due to the self-duality. We observe that the crossing point coincides exactly with the known critical point g_c (vertical dotted line), for $L_y = 4, 5, 6$ (blue, purple, orange resp.). (b) Qualitatively, we extract the phase boundary using $L_y = 4$ by finding points where the difference $|\Delta_{12}(g_0, g_1) - \Delta_{13}(g_1, g_0)| \leq 0.01$ is close to zero. (c) Along the cut of the phase diagram with g_0 small, the phase boundary computed from the difference has no visible finite size effects. (d) For larger $g_0 \geq 0.1$, small finite size effects start to appear. Shown are $g_0 = 0.1, 0.14, 0.18, 0.22, 0.26$ from left to right. The phase boundary clearly shifts to larger g_1 with increasing g_0, but $L_y = 4$ (blue) appears to slightly overestimate the transition point compared to $L_y = 6$ (orange).

Sample Complexity

A key figure of merit for certification of phases is the sample complexity, defined here as the number of samples required to confirm with 95% confidence that the measured loop operator is non-zero. To compute sample complexity, we approximate the LED Wilson loop expectation value evaluated on a large but finite size system as a Gaussian random variable. In this scenario, the important quantity is the ratio of the standard deviation σ to the mean $\langle Z_{\text{closed}}\rangle$. The estimator of the expectation value has a standard deviation that decreases as $1/\sqrt{S}$ where S is the number of samples. Thus, to confirm the mean is non-zero to two standard deviations (95% confidence), we require approximately $S = (2\sigma/\langle Z_{\text{closed}}\rangle)^2$ samples.

In Figure S2, we compare the sample complexity of certifying non-zero Wilson loops using bare and LED observables. Two scenarios are considered. In the first, bare and LED Wilson loops are compared at fixed length-scale. Indeed, the sample complexity decreases by an order of magnitude for a range of incoherent error rates below the correction threshold. In the second scenario, coarse-graining is considered, where larger length-scales are probed at each layer. Here, the sample complexity in fact increases at early layers for moderate error rates before falling dramatically. This is because the variance of the signal initially increases, since coarse-graining reduces the number of loops available for averaging in a fixed size system. However, for sufficient correction layers, LED reliably removes almost all errors. This is the regime where $\xi \ll d$, so the Wilson loops saturate at one and their variance approaches zero (see histograms in Figure S2).

Nevertheless, the initial increase in sample complexity is not simply due to information being removed. Upon further examination of the scenario without coarse-graining, we find a similar initial increase in sample complexity. We interpret this as correction causing adjacent LED loops to become correlated. Finally, we note that the sample complexity measured in this way only improves in the topological phase. In the uncorrectable, disordered phase, the inverse ratio $\langle Z_{\text{closed}}\rangle/\sigma$ rapidly approaches zero.

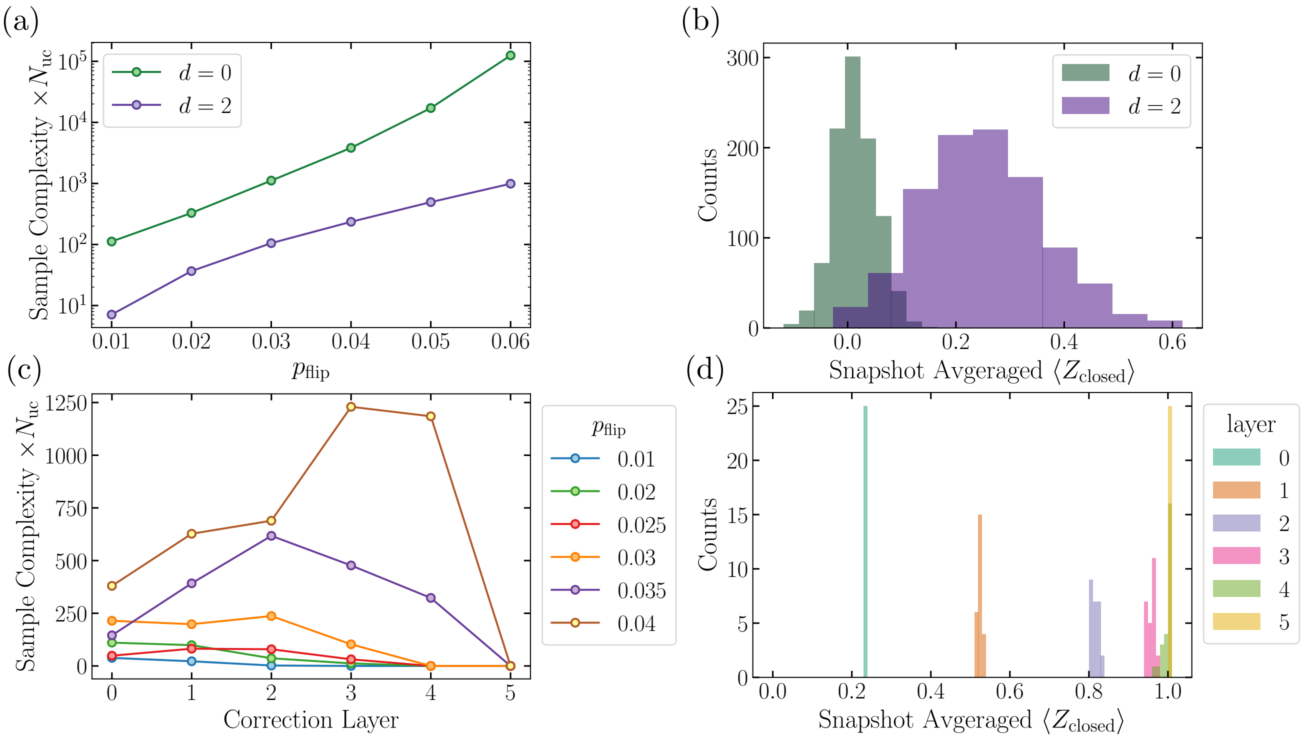

FIG. S2: Sample complexity. (a) Effect of LED on loops of fixed size $L = 10$. Snapshots are drawn from a toric code defined on a 25×25 square lattice with various bit-flip error rates. Sample complexity is computed as described in the main text. Note that sample complexities less than one imply that only a fraction of the system needs to be measured to certify topological order with 95% confidence. As such, the y-axis is also multiplied by the number of unit cells N_{uc}. (b) The histogram of expectation values averaged over a single snapshot confirms that these expectation values are approximately Gaussian distributed. The distribution for LED loops (purple) has much lower weight at zero than uncorrected loops (green), highlighting how fewer samples are needed to verify with high confidence that the closed loops are non-zero. (c,d) We also study the effect of coarse-graining on sample complexity, where the loop length $L = 5 \times 2^n$ grows exponentially with correction layer n. Here, snapshots are taken from a 1024×1024 square lattice. Initially, the sample complexity increases due to a reduction in the number of independent loops available at higher layers. However, it eventually reduces and approaches zero in the topological phase. (d) This turnaround occurs in the limit $\xi \ll d$, where correction is able to remove almost all errors, and loop expectation values approach one.

Decoder Dependence

We now examine how different choices of LED decoders can change the size of the "correctable" region—that is, the region classified as topological. The main text demonstrates results for an $l = 4$ (or $d = 2$) patch-based decoder with coarse-graining size $b = 2$; in Figure S3, we compare this with the pairing decoder and an $l = 6$ ($d = 3$) patch-based decoder which also uses $b = 2$. We see clearly that the $l = 4$ and $l = 6$ decoders both produce significantly larger correctable regions than the simple pairing decoder, for both coherent and incoherent errors. Meanwhile, the $l = 4$ and $l = 6$ decoders perform similarly, so it appears that the decoder threshold saturates with l. Intriguingly, we observe saturation at an incoherent error rate which is significantly below the known error correction threshold of $p_c \approx 10.9\%$. An interesting open question is to determine whether this discrepancy arises because the patch-based decoder is suboptimal, or because local decoders have some fundamental limit. In the Supplementary Information, we show that a "annulus-based" decoder which applies MWPM in a non-local fashion results in a much larger correctable regime.

SUPPLEMENTARY DISCUSSION

LED Circuit for $\mathbb{Z}_2$ Toric Code

In the quantum circuit formulation, the stabilizer measurement, local decoding, and coarse-graining steps of LED are implemented through local controlled-unitary gates, single-qubit rotations, and (optionally) measurements and local feed-forward operations. Meanwhile, local variational unitary gates are introduced before each LED layer to enable efficient and robust identification of a large set of topologically-ordered states (see Methods and Figure S11). The quantum circuit for stabilizer

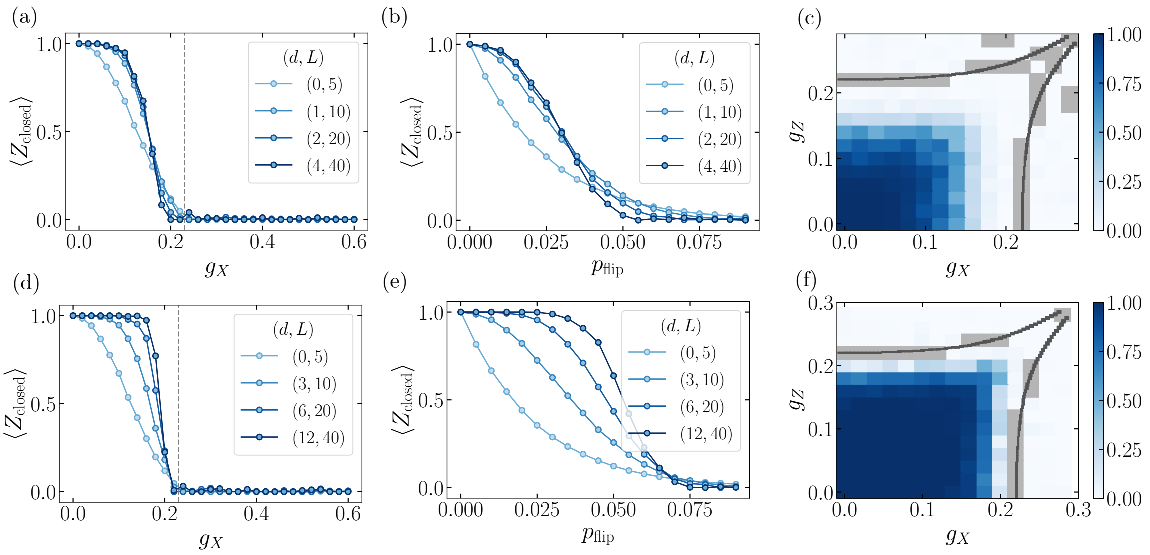

FIG. S3: Decoder choice can significantly affect the extent of the region certified as topological. Here we show LED Wilson loops using coarse-graining with block size $b = 2$ together with (a-c) a $d = 1$ pairing decoder and (d-f) a $d = 3$ patch decoder. Coherent perturbations are considered in panels (a,d), while incoherent errors are studied in (b,e). Panels (c,f) show LED expectation values for various combinations g_X,g_Z after three layers of correction. Note that in the main text, analogous plots are made using the $d = 2$ patch decoder, whose classification boundaries roughly match the $d = 3$ decoder shown here.

measurements, local decoding, and coarse-graining can be implemented in different ways, as discussed below.

As a first example, one can introduce an ancillary qubit for each stabilizer, and perform local controlled-NOT (CNOT) gates between the system qubits and the ancillary qubit at each vertex or plaquette. These local CNOT gates are designed in the exact same fashion as stabilizer measurements of the surface code (see, for example, Ref. [3]). Decoding can then be implemented either by measuring the ancillary qubits and performing local feed-forward operations on the system qubits to correct for arbitrary single-qubit errors, or by using local controlled-unitary operations between the ancillary and system qubits to achieve the same result. Finally, the MERA circuit of Ref. [4] can be used to perform coarse-graining at each LED layer.

Alternatively, one can avoid introducing ancillary qubits by carefully constructing a circuit which maps stabilizer values in each layer to the qubits which are removed during the coarse-graining process of that layer. This scheme combines the stabilizer measurement and coarse-graining procedures together into one large set of unitary operations. As before, the decoding step can be implemented by measuring stabilizer qubits and performing local feedforwarding to the remaining qubits, or by using local controlled-unitary operations between the ancillary and system qubits.

Because the number of removed qubits in each unit cell is always smaller than the number of stabilizers in this second case, only a fraction of stabilizers can be measured in every layer. Thus, one must design the circuit meticulously in order to still correct for all single-qubit errors. As a concrete example, we illustrate here such a quantum circuit implementation of the LED stabilizer measurement, local decoding, and coarse-graining procedures for the $\mathbb{Z}_2$ toric code which does not require additional ancillary qubits.

In this circuit, nine unit cells are combined to a single, large unit cell in the next layer by the coarse-graining procedure (i.e., a 3-to-1 reduction is performed in each dimension). The circuit is invariant under translation by the large unit cells, so we consider only the gates involving a single large unit cell. The setup and notations for qubits are defined in Figure S4.

Our circuit consists primarily of two-qubit CNOT gates, together with some single-qubit rotations and multi-qubit controlled-unitary operations. Thus, to compactly specify our circuit, we introduce some notations for the controlled-unitary operations of our circuit: specifically, we use the notation (control qubits $\mapsto$ target qubit) to refer to the gate which performs a bit-flip (X) gate on the target qubit if and only if all control qubits are in the $Z = -1$ state, and identity otherwise. For example, $(a \mapsto b)$ denotes a CNOT gate where a is the control qubit and b is the target qubit. Likewise, $(a, b \mapsto c)$ denotes a Toffoli gate where a and b are the control qubits and c is the target qubit.

With all notations defined, we are now ready to specify our circuit. Due to translation invariance, it is understood that whenever we list a gate here, it will also be applied simultaneously on all qubits which differ from the labeled sites by translation of an integer number of

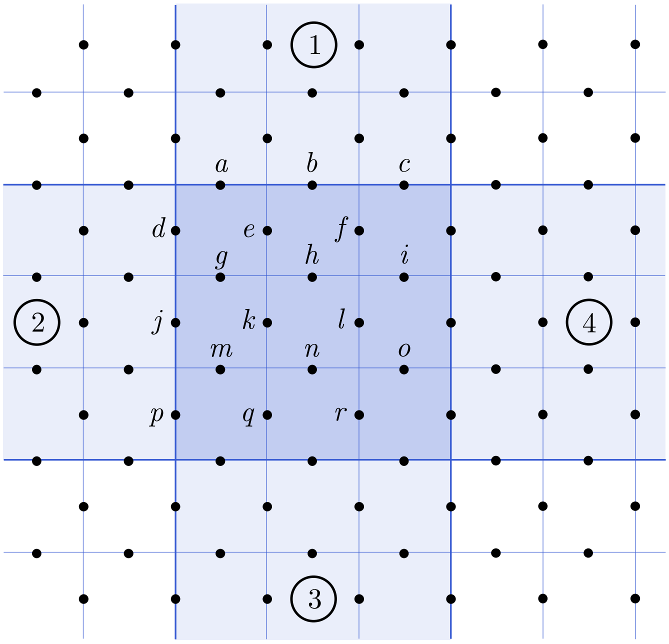

FIG. S4: Setup for a quantum circuit which implements LED for the $\mathbb{Z}_2$ toric code phase without introducing any ancillary qubits. The coarse-graining performed by this circuit combines nine unit cells into a single, large unit cell in the subsequent layer (thick vs. thin lines); we label each of the 18 qubits in this large unit cell using the letters a through r as shown above. Here, we focus on the local unitaries involving the qubits within a single, large unit cell (darker blue shaded cell); however, some of the local unitaries in our circuit span two large unit cells, so we also consider the neighboring cells (lighter blue shaded cells). We label the neighboring cells 1 through 4, and refer to a qubit in such a cell by its letter and the number of the cell (e.g., the uppermost qubits of unit cell 2 would be a_2, b_2, and c_2 from left to right).

large unit cells; thus, our circuit consists of many layers of non-overlapping local gates. The circuit proceeds as follows:

1. Perform layers of CNOT gates in the following order: $(i \mapsto o)$, $(l \mapsto o)$, $(j_4 \mapsto o)$, $(f \mapsto i)$, $(c \mapsto i)$, $(d_4 \mapsto i)$, $(b \mapsto f)$, $(e \mapsto f)$, $(h \mapsto f)$, $(a \mapsto e)$, $(d \mapsto e)$, $(g \mapsto e)$, $(j \mapsto g)$, $(m \mapsto g)$, $(k \mapsto g)$, $(p \mapsto m)$, $(a_3 \mapsto m)$, $(q \mapsto m)$, $(q \mapsto n)$, $(b_3 \mapsto n)$, $(r \mapsto n)$, $(q \mapsto k)$, $(r \mapsto l)$, $(b_3 \mapsto h)$, $(k \mapsto h)$, $(l \mapsto h)$, $(a \mapsto b)$, $(c \mapsto b)$, $(d \mapsto j)$, $(p \mapsto j)$.

2. Perform layers of Toffoli gates in the following order: $(e, m_1 \mapsto b)$, $(f, n_1 \mapsto b)$, $(i_2, e \mapsto j)$, $(g, o_2 \mapsto j)$.

3. Perform single-qubit X gates on e, f, g, h, n, o.

4. Perform two layers of multi-qubit controlled-unitary gates $(i, e_4, f, o \mapsto b)$, $(m, e_3, g, h \mapsto j)$

5. Perform single-qubit Hadamard gates on a, b, c, d, j, k, l, p.

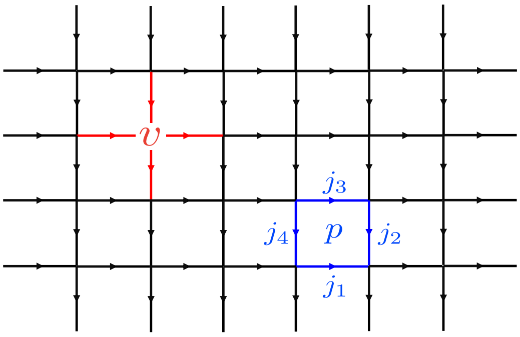

FIG. S5: Setup for Kitaev's quantum double models on a square lattice [5]. For generic groups G, one must define an orientation for each edge; for simplicity, we choose edges to be oriented upwards and to the right.

6. Perform layers of Toffoli gates in the following order: $(a, c \mapsto b)$, $(d, k \mapsto j)$, $(c_3, l \mapsto b)$, $(c_3, l \mapsto j)$, $(c_3, l \mapsto b_3)$, $(d, p \mapsto j)$, $(r, c_3 \mapsto b_3)$, $(r, p_4 \mapsto b)$, $(r, p_4 \mapsto j)$, $(r, p_4 \mapsto j_4)$.

7. Perform single-qubit Hadamard gates on b and j.

LED for Abelian Quantum Double Models

The snapshot-based LED framework can be extended to enable detection of Kitaev's quantum double models based on any finite abelian group G. Since the quantum double model $\mathfrak{D}(G)$ of a direct product of finite groups $G = G_1 \times G_2 \times ... \times G_k$ is equivalent to the model where k quantum doubles $\mathfrak{D}(G_1)$, ..., $\mathfrak{D}(G_k)$ are stacked together, it suffices to consider the quantum double of cyclic groups $G_d = \{0, 1, ..., d-1\}$. In such models, an orientation must be assigned to each link of the square lattice, which is occupied by a *qudit* belonging to the Hilbert space spanned by $\{|j\rangle, j \in G_d\}$ (Figure S5). To define the vertex and plaquette stabilizers for these quantum double models, we first introduce the single-qudit *shift* and *clock* operators—$X_\pm$ and $Z_\pm$, respectively—which are generalizations of the Pauli matrices X and Z for qubits. We define these operators based on their action on the basis states:

$$X_\pm |j\rangle = |j \pm 1\rangle \tag{1}$$

$$Z_\pm |j\rangle = \omega^j |j\rangle. \tag{2}$$

where addition is performed modulo d, and $\omega = e^{2\pi i/d}$ is a dth root of unity. Using these operators, we can define generalized vertex and plaquette operators:

$$A(v) = \prod_{j \in \text{adj}(v)} X(j, v) \tag{3}$$

$$B(p) = \prod_{j \in \text{adj}(p)} Z(j, v). \tag{4}$$

Here, the X and Z operators depend on the orientation of the edge j relative to the vertex v or plaquette p under consideration: if the directed edge j points away from v, $X(j,v) = X_-(j)$, otherwise $X(j,v) = X_+(j)$; if p is on the left (resp., right) of the directed edge j when the lattice is rotated such that j points upwards, $Z(j,p) = Z_-(j)$ (resp., $Z_+(j)$) [5]. The Hamiltonian for this model is then

$$H = -\sum_v \sum_{k=0}^{d-1} A(v)^k - \sum_p \sum_{k=0}^{d-1} B(p)^k. \tag{5}$$

As in the case of the toric code Hamiltonian (Equation (**??**) in the main text), all $A^k(v)$ and $B^k(p)$ terms in the above Hamiltonian H commute with each other. Each $A(v)$ and $B(p)$ has d possible eigenvalues: 1, ω, ω^2, ..., ω^{d-1}. The ground state(s) of H are then simultaneous $+1$ eigenstate(s) of all $A(v)$ and $B(p)$. Meanwhile, vertex and plaquette violations correspond to anyonic excitations: a plaquette where $B(p) = \omega^a$ hosts an e^a anyon, while a vertex where $A(v) = \omega^b$ hosts an m^b anyon. Thus, at any site (v,p), there are d^2 possible topological charges $e^a m^b$ $(a, b \in \{0, 1, ..., d-1\})$.

We now demonstrate how a generalized snapshot-based LED procedure can be used to recognize the quantum double phase. In this case, we begin by measuring all qudits in the basis $\{|0\rangle, |1\rangle, ..., |d-1\rangle\}$, which we refer to as the *group basis*. Such a measurement allows us to compute all plaquette terms $B(p)$, and identify e-type anyons. The location of such an anyon can be shifted one cell away by applying $X_\pm^{B(p)}$ to one of the edges in $\mathrm{adj}(p)$ (the sign depends on the edge's orientation relative to the plaquette): for example, if the anyon is located on the plaquette p in Figure S5, applying $X_-^{B(p)}$ to j_3 will move the anyon up by one cell. The patch-based decoder we use for LED thus corrects errors by grouping together, when possible, two or more plaquettes $p_1, p_2, ...$ with nontrivial $B(p_i)$ such that the product $\prod_i B(p_i) = 1$; this can be implemented simply by multiplying qudits by group elements $B(p_i)^{\pm 1}$. The groupings are chosen to minimize the total number of qudits to modify, while still removing as many errors as possible within each patch.

The above procedure allows us to correct for e-type errors. The same LED procedure can be performed to address m-type errors, by measuring qudits in another basis—the *representation basis*. Representation-basis measurements are performed by first applying a generalized Hadamard operator

$$H_d = \frac{1}{\sqrt{d}} \sum_{a=0}^{d-1} \sum_{b=0}^{d-1} \omega^{ab} |a\rangle\langle b|, \tag{6}$$

and then measuring in the group basis. Because the shift operator is diagonal in the representation basis, measurements in this basis allow us to identify m-type anyons, and utilize the patch-based decoding scheme described above for LED.

Arbitrary Local Perturbations

In this section, we show that LED Wilson loops flow to one for any state which differs from the fixed-point state by an arbitrary local perturbation. This implies that LED loop operators are independent of the exact perturbation, unlike the fattened Wilson loops of Refs. [6, 7]. For concreteness, we examine perturbations on top of a toric code ground state.

To prove our claim, we consider a local unitary operator $\mathcal{O}$ supported on a local region A of diameter l. It follows that $\mathcal{O}$ can only flip a stabilizer from $+1$ to -1 if it overlaps with A, and $\mathcal{O}$ cannot couple any ground state $|\psi\rangle$ to another ground state $|\psi'\rangle \neq |\psi\rangle$. We now show that LED removes all flipped stabilizers after $1 + \log_b d$ layers, where b is the coarse-graining length-scale. Because the coarse-graining step effectively reduces $l \to l/b$, after $\log_b d$ layers, there are three possibilities: (1) A becomes fully contained within a single $b \times b$ region at some layer $c < \log_b d$. Then, A has zero support after another layer of coarse-graining and disappears. (2) Before iteration $\log_b d$, $\mathcal{O}$ is supported on two adjacent $b \times b$ regions. Then, $\mathcal{O}$ becomes a single-qubit error after this iteration and is removed by the subsequent LED step. (3) Before iteration $\log_b d$, $\mathcal{O}$ is supported at the corner of three or four regions. In this case, it becomes a two-qubit diagonal error after this iteration, which can also be removed by the subsequent LED step. Notice that handling case (3) requires the inclusion of diagonal pairing in the pairing decoder. Finally, while this proof focuses on the pairing decoder, it also generalizes directly to more advanced local decoders, such as the patch-based decoder, when they are combined with coarse-graining.

Proof of Theorem 1

Theorem 1. *Let $|\psi\rangle$ be an arbitrary input state defined on a surface with trivial topology. Then, after performing LED with correction distance d, assume the resultant state $|\psi_d\rangle$ has, as a subsystem, qubits living on the links of a square lattice, as in the toric code. Then, if the stabilizer expectation values $\langle \frac{1+A_v}{2} \rangle = \left\langle \frac{1+B_p}{2} \right\rangle = 1$ at every vertex v and plaquette p of the subsystem, then, the input state $|\psi\rangle$ is topologically-ordered, in the sense that it is connected to an output state of the form $|\psi_d\rangle = |\psi_{\mathrm{TC}}\rangle \otimes |\phi_{\mathrm{anc}}\rangle$ by generalized local unitary (gLU) transformation of depth $O(d)$.*

Proof. The LED procedure forms a local quantum channel, and we begin our proof by constructing a purification of this channel. To mediate stabilizer measurement and local error correction, one can first introduce an ancilla in the state $|0\rangle$ at every vertex and plaquette. Next, a sequence of Hadamard and controlled-NOT (CNOT) gates is applied such that a Z-basis measurement on an ancilla

is equivalent to the associated stabilizer measurement of A_v or B_p. Then, local quantum error correction is performed using a local unitary evolution on the combined system, which contains the original state and the added ancilla qubits. This local unitary evolution applies gates which perform X and Z spin flips on the system qubits, conditioned on the state of the ancilla qubits. Finally, the coarse-graining step can also be performed with local unitary transformations by using a quantum circuit corresponding to a multiscale entanglement renormalization ansatz (MERA) representation of the fixed-point state [4]. The transformations generated by introducing product state ancillas and performing local unitary operations are called generalized local unitaries (gLU); this class of transformations includes our LED procedure described above and is known to preserve phase boundaries [8].

If the system part of the final state, $|\psi_d\rangle \in \mathcal{H}_{\rm sys}\otimes\mathcal{H}_{\rm anc}$, has stabilizer expectation values $\left\langle \frac{1+A_v}{2}\right\rangle = \left\langle \frac{1+B_p}{2}\right\rangle = 1$ at every vertex v and plaquette p, then $|\psi_d\rangle$ must belong to the ground state space of the toric code. This is because the projector onto the ground state space is given by the product of all the stabilizers $P_{\rm TC} = \prod_v \frac{1+A_v}{2}\prod_p \frac{1+B_p}{2}$. On a surface with trivial topology, there is a unique state $|\psi_{\rm TC}\rangle$, so the output state factors into $|\psi_d\rangle = |\psi_{\rm TC}\rangle \otimes |\phi_{\rm anc}\rangle$. □

Proof of Lemmas 3 and 4

Lemma 3. *Given an output state $|\psi_d\rangle$ satisfying the conditions of Theorem ??, and a simply connected $(\mathscr{L}-2)\times(\mathscr{L}-2)$ square region R on the system part, the reduced density matrix $\rho_d = \mathrm{Tr}_{R^c}[|\psi_d\rangle\langle\psi_d|]$ is indistinguishable from the toric code reduced density matrix $\sigma_{\rm TC} = \mathrm{Tr}_{R^c}[|\psi_{\rm TC}\rangle\langle\psi_{\rm TC}|]$ defined on the same region, up to the bound $||\rho_d-\sigma_{\rm TC}|| \le \max\left(\sqrt{\epsilon}, 2\mathscr{L}^2\epsilon\right)$*

Proof. To bound the trace distance, we will use the fact that our state ρ_d locally looks almost the same as the toric code state. Specifically, trace distance is related to distinguishability by [9]

$$||\rho-\sigma|| = \frac{1}{2}\sup_{||O||\le 1}\mathrm{Tr}\left[O(\rho-\sigma)\right]. \qquad (7)$$

To upper bound this, we can consider all possible unit norm operators O. Specifically, O can always be written as a linear combination of Pauli strings. These Pauli strings can be analyzed by considering two cases, closed strings and open strings. First consider operators C supported on R, which commute with all stabilizers A_v, B_p supported on a slightly larger $\mathscr{L}\times\mathscr{L}$ region (1-ball or R) , constructed by expanding on all sides by one unit cell. These operators must be products of contractible Wilson loops, and hence can be written as product of stabilizers. Therefore, $\mathrm{Tr}[\sigma_{\rm TC}C] = 1$ for the toric code state. For the LED output state, we instead bound the expectation value of $P_{\rm TC} = \prod_v \frac{1+A_v}{2}\prod_p\frac{1+B_p}{2}$, where the product over v (resp., p) runs over all vertices (plaquettes) within the $\mathscr{L}\times\mathscr{L}$ region. The expectation value of this projector, evaluated on the output state, is given by $\langle\psi_d|P_{\rm TC}|\psi_d\rangle$. To lower bound this quantity, we first notice that every term in $P_{\rm TC}$ has spectrum $\{0,1\}$, and that the terms are mutually commuting.

This allows us to approximate $\langle P_{\rm TC}\rangle$ using the individual expectation values $\left\langle\frac{1+A_v}{2}\right\rangle > 1-\epsilon$ and $\left\langle\frac{1+B_p}{2}\right\rangle > 1-\epsilon$. To do so, we note that if two commuting operators A and B, each with spectrum $\{0,1\}$, satisfy $\langle A\rangle > 1-\epsilon$ and $\langle B\rangle > 1-\epsilon$, then $\langle AB\rangle > 1-2\epsilon$. To show this, we add the two individual bounds to obtain $\langle A\rangle + \langle B\rangle > 2-2\epsilon$; moreover, since AB has spectrum $\{0,1\}$, we have $\langle A\rangle+\langle B\rangle-\langle AB\rangle < 1$. It thus follows that $\langle AB\rangle > 1-2\epsilon$, and upon applying this recursively to include all vertex and plaquette terms within the $\mathscr{L}\times\mathscr{L}$ region, we obtain $\langle\psi_d|P_{\rm TC}|\psi_d\rangle > 1-2\mathscr{L}^2\epsilon$.

Using this fact, along with $CP_{\rm TC} = P_{\rm TC}$ we can similarly lower bound the expectation value $\mathrm{Tr}[\rho_d C] \ge 1-4\mathcal{L}^2\epsilon$. Thus, for any C, we have $\frac{1}{2}|\mathrm{Tr}\left[C(\rho_d-\sigma_{\rm TC})\right]| \le 2\mathcal{L}^2\epsilon$.

Next, we consider operators O which anti-commute with some stabilizers. In particular, these operators are Pauli strings with at least one endpoint (open strings). Naturally, their expectation value vanishes in the toric code state. To see this, let S be one of the stabilizers which anti-commutes with O. Now, S and O form an anti-commuting pair of Pauli operators, so they satisfy an uncertainty relation $\langle S\rangle^2 + \langle O\rangle^2 \le 1$. As such, in the toric code where $\langle S\rangle = 1$, this implies $\langle O\rangle = 0$. Similarly, the condition $\mathrm{Tr}[\rho_d S] \ge 1-2\epsilon$ leads to the upper bound $\mathrm{Tr}[\rho_d O] \le 2\sqrt{\epsilon}$. Combining these two results, we see the trace distance is at most $T(\rho_d,\sigma_{\rm TC}) \le \max\left(\sqrt{\epsilon}, 2\mathscr{L}^2\epsilon\right)$. □

Lemma 4. *Consider an input state ρ and an LED procedure satisfying the conditions of Theorem ??. Then the final state $|\psi_d\rangle$ after LED cannot be prepared using a local quantum circuit with depth less than $O(\mathscr{L}) \sim O(1/\sqrt{\epsilon})$.*

To prove these results, we extend and generalize the proof techniques developed by Ref. [10]. Note that for notional simplicity, here we work with an input state $|\psi\rangle$ that is a purification of ρ. This is done without loss of generality, since all of the operations act on the original degrees of freedom.

Consider a pair of Z-basis and X-basis Wilson loops A and B, supported on two overlapping annuli. The twist product of the two operators $A\infty B$ is defined such that at one intersection region, operator B is applied first, while at the other operator A is applied first (Fig. S6). By the arguments of Ref. [10], such a pair of locally non-commuting observables, whose twist product does not factorize into a product of the individual observables, can

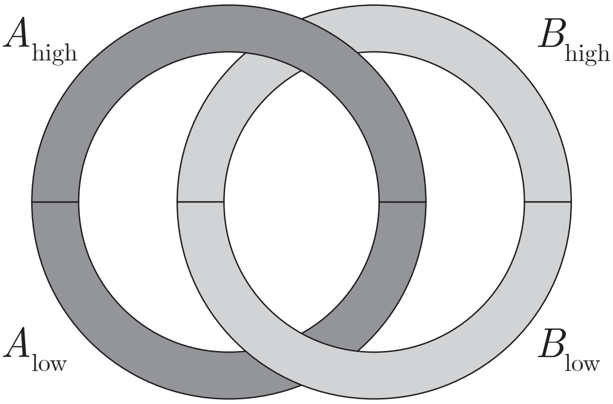

FIG. S6: Consider two operators A and B, each supported on an annulus which intersects at two regions, one higher and one lower. The twist product $A \infty B$ consists of applying B first in the higher region, and A first in the lower region (also see Ref. [10] Fig. 1 and Eq. 4). In the diagram the order of operations goes back to front. Furthermore, for tensor product operators which can be written as $A = A_{\text{low}} A_{\text{high}}$, $B = B_{\text{low}} B_{\text{high}}$, we can write the twist product as $A \infty B = B_{\text{low}} A_{\text{high}} A_{\text{low}} B_{\text{high}}$.

serve as a witness for long-range entanglement. In our case, since Z- and X-strings locally anti-commute, we can remove the twist to get $A \infty B = -AB$.

Then, Ref. [10] showed that, assuming the observables A and B satisfy an additional important property called local invisibility (see below), then the following correlation serves as a witness for long-range order.

$$|\langle\psi| A \infty B |\psi\rangle - \langle\psi| A |\psi\rangle \langle\psi| B |\psi\rangle| > 0. \tag{8}$$

Specifically, the state $|\psi\rangle$ cannot be prepared from a trivial state by a circuit of depth $O(L)$, where L is the separation between the two intersection regions. Indeed, in the exact case, where the expectation value of large LED Wilson loops are one, the results of Ref. [10] can be directly applied.

However, the proofs in Ref. [10] do not immediately apply to the realistic case considered here, where stabilizers have expectation value $1 - \epsilon$, and residual entanglement between the system and ancilla or environment qubits prevents exact knowledge of the state. Nevertheless, with sufficient care and a few additional assumptions, approximate versions of key results in Ref. [10] can be recovered.

First, we develop a notion of *approximate local invisibility*. Throughout, we follow the spirit of the proofs in Section III of Ref. [10]; the reader is encouraged to consult the original reference for additional details and insights.

Definition 1 (Approximate (Δ, r, t)-local invisibility.)**.** Let A be a region of radius r and B be a t-ball around A. An operator O with unit norm is (Δ, r, t)-locally invisible with respect to a state $|\psi\rangle$ if, for any state $|\phi\rangle$ whose reduced density matrix on B is equivalent to $|\psi\rangle$, it satisfies

$$\left|\left| \frac{\mathrm{Tr}_{A^c}[O |\phi\rangle \langle\phi| O^\dagger]}{\mathrm{Tr}[O |\phi\rangle \langle\phi| O^\dagger]} - \mathrm{Tr}_{A^c}[|\psi\rangle \langle\psi|] \right|\right| \le \Delta, \tag{9}$$

where the norm is the standard trace norm. In other words, locally invisible operators leave local reduced density matrices approximately unchanged. Note that we restrict to states $|\phi\rangle$ for which the expectation value does not vanish, such that this remains well-defined. This subtlety is also present in the original definition of Ref. [10].

Next, we will show that Wilson loops that nearly stabilize $|\psi_d\rangle$ are approximately locally invisible. Let A be a region of radius r, which can only cover a patch of the loop. Furthermore, let $t = 0$, i.e. region B is identical to region A. Since the Wilson loop is a tensor product of local unitaries, we can write $O = O_B \otimes O_{B^c}$ and $O^\dagger_{B^c} O_{B^c} = 1$. This allows us to work directly with the reduced density matrices of region B, and we can use Lemma 3 to reduce to the toric code case

$$\mathrm{Tr}_{A^c}[O\rho_d O] = \mathrm{Tr}_{A^c}[O\sigma_{\mathrm{TC}} O] + \mathrm{Tr}_{A^c}[O(\rho_d - \sigma_{\mathrm{TC}})O] \tag{10}$$

Indeed, since O is locally invisible with respect to to the toric code, this gives us our result, where the error term depends on the size of B.

$$||\mathrm{Tr}_{A^c}[O\rho_d O] - \mathrm{Tr}_{A^c}[\sigma_{\mathrm{TC}}]|| \le \max\left(\sqrt{\epsilon}, 2(r+1)^2\epsilon\right) \tag{11}$$

More microscopically, O spans the region, so locally looks like a logical operator. The reduced density matrix σ_{TC} on region B is an equal weight mixture of all logical states, so O leaves it invariant.

This shows that Wilson loops are (Δ, r, t)-locally invisible with respect to $|\psi_d\rangle$ for $t \ge 0$ and $\Delta = \max\left(\sqrt{\epsilon}, 2(r+1)^2\epsilon\right)$. When combined with the fact that Wilson loops have large expectation value on $|\psi_d\rangle$, this will serve as a witness for long-range topological order.

To prove this, we need to confirm that, even for the weaker notion of approximate local invisibility, the twist product approximately factorizes for trivial states.

Lemma 2. *The twist product of two (Δ, r, t) locally invisible operators A and B, acting on a trivial product state $|\psi\rangle = |00...0\rangle$, must satisfy*

$$|\langle\psi| A \infty B |\psi\rangle - \langle\psi| A |\psi\rangle \langle\psi| B |\psi\rangle| \le O(\sqrt{\Delta R/r}) \tag{12}$$

where A and B are supported on two annuli which intersect at two regions (Fig. S6) whose separation is $\ge 2(r+t)$.

Proof. In the first step of the proof, we bound the expectation value of $\Pi_{\mathcal{R}} = \prod_{i \in \mathcal{R}} |0\rangle \langle 0|_i$, the projector onto $|\psi\rangle$

supported on region $\mathcal{R}$ evaluated with respect to $O|\psi\rangle$ for unitary O.

Invoking the definition of local invisibility, we show for A of radius r, that $\langle\psi| O^\dagger \Pi_A O |\psi\rangle \geq 1-\Delta$.

$$\left|\left|\mathrm{Tr}_{A^c}\left[O|\psi\rangle\langle\psi|O^\dagger\right] - \Pi_A\right|\right| \leq \Delta \tag{13}$$

Thus, the expectation value of the observable Π_A satisfies:

$$|\mathrm{Tr}\left[\Pi_A O|\psi\rangle\langle\psi|O^\dagger\right] - 1| \leq \Delta \tag{14}$$

$$\langle\psi| O^\dagger \Pi_A O |\psi\rangle \geq 1-\Delta \tag{15}$$

Next, we can use the fact that $\Pi_{\mathcal{R}}$ can be written as a product of Π_A approximately R/r times. It follows from the same union bound argument in Lemma 3, that $\langle\psi| O^\dagger \Pi_{\mathcal{R}} O |\psi\rangle \geq 1-\Delta R/r$. This result can be used to bound the distance between the two states,

$$||\Pi_{\mathcal{R}} O|\psi\rangle - O|\psi\rangle||^2 \leq \Delta R/r, \tag{16}$$

by noticing the left side is equal to $1 - \langle\psi| O \Pi_{\mathcal{R}} O |\psi\rangle$. We will use this below, to show that we can replace $O|\psi\rangle$ with $\Pi_{\mathcal{R}} O|\psi\rangle$ without incurring significant error.

To prove the main result, we use the same construction as Ref. [10]. Specifically, we want to use the above result to show that $A \infty B|\psi\rangle = (A\Pi_{\mathcal{R}})\infty B|\psi\rangle + O(\Delta\mathcal{L}^2)$. We do this by carefully inserting projectors. For Wilson loops, which are tensor product operators, we the twist product can be split up as follows (see Fig. S6),

$$A \infty B = B_{\mathrm{low}} A_{\mathrm{high}} A_{\mathrm{low}} B_{\mathrm{high}}. \tag{17}$$

Our above result implies $\Pi_{\mathcal{R}} B_{\mathrm{high}}|\psi\rangle = B_{\mathrm{high}}|\psi\rangle + O(\Delta R/r)$. We can subsequently pull projectors from $|\psi\rangle$ to cover the region of support of A, e.g. low and the parts of high. Thus, we get

$$\begin{aligned} A\infty B|\psi\rangle =& B_{\mathrm{low}} A_{\mathrm{high}} \Pi_{\mathrm{high}} A_{\mathrm{low}} \Pi_{\mathrm{low}} B_{\mathrm{high}}|\psi\rangle \\ &+ O(\sqrt{\Delta R/r}) \\ =& (A\Pi)\infty B|\psi\rangle + O(\sqrt{\Delta R/r}) \end{aligned} \tag{18}$$

as we wanted. Finally, we this implies the expectation value of the twist product approximately factorizes $\langle\psi| A\infty B|\psi\rangle = \langle\psi| A|\psi\rangle\langle\psi| B|\psi\rangle + O(\sqrt{\Delta R/r})$. □

Although we proved that the twist product must factorize for the trivial product state, this holds for a much wider class of short-range entangled states, generated from a trivial state by a finite-depth unitary circuit. In particular, using the same argument as Haah Lemma III.3 shows that, given a (Δ, r, t)-locally invisible operator O and state $|\psi\rangle$, if we evolve under a local unitary circuit W of depth d, then the operator $WOW^\dagger$ is $(\Delta, r-d, t+2d)$-locally indistinguishable with respect to $W|\psi\rangle$. This will be used to show that non-factorizability of the twist product lower bounds the depth of a quantum circuit required to produce the state from the trivial product state.

Finally, we can use the trace distance Lemma 3, to show the twist product does not factorize for $|\psi_d\rangle$. Specifically, let A and B be Wilson loops supported on an $\mathscr{L} \times \mathscr{L}$ region. Then,

$$|\langle\psi_d| A\infty B|\psi_d\rangle - \langle\psi_d| A|\psi_d\rangle\langle\psi_d| B|\psi_d\rangle| \geq 2 - c\mathscr{L}^2\epsilon \tag{19}$$

for a constant c. Combining this Lemma 6 for trivial states, we see the bound is violated when

$$2 - c\mathscr{L}^2\epsilon > c'\sqrt{\Delta\mathscr{L}/r} \tag{20}$$

$$2 - c\mathscr{L}^2\epsilon - c' 2(r+1)\sqrt{\mathscr{L}\epsilon/r} \geq 0 \tag{21}$$

We choose r to be a constant fraction of $\mathscr{L}$, and see that we can roughly make $\mathscr{L} \sim O(1/\sqrt{\epsilon})$ and still certify long-range order. In particular, the state $|\psi_d\rangle$ cannot be generated by a finite depth circuit of depth smaller than $r \sim O(\mathscr{L})$. Since $|\psi_d\rangle$ is connected to the input state by a depth-d quantum circuit, this implies LED Wilson loops close to one certify topological order up to length-scales $O(\mathscr{L} - d)$.

Connection Between LED and Entanglement Negativity

The LED framework suggests a characterization of topological order based on the ability to distill the fixed-point wavefunction using generalized local unitary operations. In this section, we connect this definition to the topological entanglement negativity of the input state, a typical observable used to detect topological order in mixed states [11, 12].

As described above, the circuit construction for LED involves applying local unitary transformations to the input mixed state ρ_S and product state ancillas $|0\rangle_A\langle 0|$. States that are classified as topological by LED are connected via a finite-depth local unitary to the toric code fixed-point state and an ancillary register (see Theorem **??**).

$$\rho_S \otimes |0\rangle_A\langle 0| \to |\psi_{\mathrm{TC}}\rangle_{S'}\langle\psi_{\mathrm{TC}}| \otimes \sigma_{A'}$$

In particular, all of the entropy in the input state, associated with both incoherent and coherent fluctuations away from the fixed point state, is transferred to the ancilla $\sigma_{A'}$.

We conjecture that the ability to "distill" the toric-code fixed point state also implies the presence of a topological correction to the entanglement negativity of the input state. Although we cannot prove this statement rigorously, we make the connection more precise in this section. First, we make the plausible assumption that

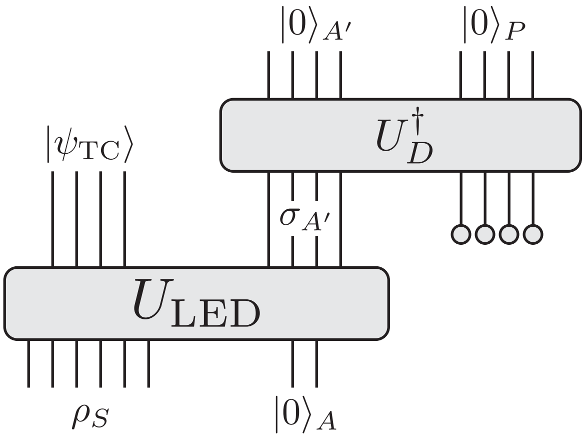

FIG. S7: Theorem 1 shows that a positive LED classification implies the input mixed state ρ_S and product state ancilla's (A) can be connected to an output state of the form $|\psi_{\rm TC}\rangle_{S'}\langle\psi_{\rm TC}|\otimes\sigma_{A'}$, where S' is typically smaller than S due to coarse-graining. To make a connection between LED and entanglement negativity of ρ_S, we further assume $\sigma_{A'}$ is trivial. In particular, we assume there is a local purification of $\sigma_{A'}$ that is connected to the product state via local unitaries. Equivalently, we assume $\sigma_{A'}$ can be generated from a product state by applying local unitaries and tracing out some degrees of freedom.

the ancilla $\sigma_{A'}$ is in a trivial mixed state. This ensures all of the topological contribution to the entanglement negativity comes from the system part S'. Then, we argue the topological contribution remains after applying inverse LED circuits (coarsening of the Wilson loops). In particular, if we consider local Clifford circuits, we can show this is indeed the case. Together, these arguments suggest that the topological order witness provided by LED should also serve as a witness for other quantities like entanglement negativity in many scenarios of practical interest.

The key assumption we make is that the ancilla $\sigma_{A'}$ is in a trivial mixed state. Concretely, we assume that there exists a *local purification* of $\sigma_{A'}$ which is connected to a product state under local unitary circuits (Figure S7). This local purification is constructed by doubling the number of qubits, such that $\sigma_{A'}={\rm Tr}_P[|\Sigma\rangle\langle\Sigma|]_{A'P}$. Here, P is the subsystem which implements the purification. Qubits in P are placed next to qubits in A', so that the local structure is preserved. Then, we assume that there is a local unitary $U_D^\dagger$ which maps $|\Sigma\rangle$ to a product state. Thus, $\sigma_{A'}={\rm Tr}_P[U_D|0\rangle\langle 0|U_D^\dagger]$ can be "locally purified" to a product state. We note that in the circuit model, the assumption about $\sigma_{A'}$ may be checkable via state tomography.

Taking into account the purified register of qubits, the output state can now be written as a pure state. Further, it is a stabilizer state, with toric code stabilizers A_v, B_p for the S' subsystem, and trivial stabilizers Z_i for the combined $A'P$ subsystem. Note we assume for simplicity a topologically trivial manifold, so there are no non-trivial logical operators. Our construction then implies that this stabilizer state is connected to a purification of the input state via local unitary circuits. If we then trace out the degrees of freedom associated with P, this produces the input mixed state. We can leverage the stabilizer structure to make concrete statements about the entanglement negativity of this input mixed state.

First, we review the calculation of the entanglement negativity from Ref. [13], for the toric code output state. Consider a partition of the system into region R and its complement R'. Note that R is associated with a region of space, and hence contains sites from all three subsystems S', A', and P. The entanglement negativity is given by $S_R=\log||(\rho')^{T_A}||_1=\log(\sum_i p_i')$, where T_A is the partial transpose of the output density matrix, and p_i' are the associated eigenvalues. The partial transpose of the density matrix ρ, which is defined as

$$\rho = \sum_{i,i',j,j'} \rho_{i,i',j,j'}|i\rangle_A\langle i'|\otimes|j\rangle_{A^c}\langle j'| \tag{22}$$

$$\rho^{T_A} = \sum_{i,i',j,j'} \rho_{i,i',j,j'}|i\rangle_A\langle i'|\otimes|j'\rangle_{A^c}\langle j| \tag{23}$$

where $|i\rangle_A$ and $|j\rangle_{A^c}$ form an orthonormal basis for the two parts of the system. To start, notice that the only non-zero eigenvalue of the bare output state ρ' corresponds to the simultaneous $+1$ eigenstate of all the stabilizers, A_v, B_p, and Z_i.

Under the inverse LED operations, each individual stabilizer is coarsened, making exact computation of the entanglement negativity difficult. However, the LED circuit provides additional structure. Importantly, the toric code stabilizers map onto the system part. These become the LED Wilson loops which we measure in the input state. In contrast, the trivial stabilizers can also map onto subsystem that will be traced out. As a result, we expect the coarsened toric code stabilizers will still contribute a topological term in the entanglement negativity. We conjecture this is true for any local unitary circuit. However, we are able to calculate it if we consider the specific case of local Clifford circuits. Note that the LED circuits which implement local pairing decoders are not Clifford circuits, and hence the argument does not rigorously apply in that case.

Let A_v, B_p label the toric code stabilizers in the original state, and Z_i the stabilizers of the ancilla. Under the Clifford unitary, these stabilizers are mapped to coarsened versions, $\tilde{A}_v, \tilde{B}_p, \tilde{Z}_i$.

The reduced density matrix of the input state, before

tracing out the auxilliary qubits, can be written as

$$\rho = \prod_v \frac{1+\tilde{A}_v}{2} \prod_p \frac{1+\tilde{B}_p}{2} \prod_i \frac{1+\tilde{Z}_i}{2}. \quad (24)$$

Suppose there are $N = V + P + A$ stabilizers and an equivalent number of qubits. This is the case if we are working on a topologically trivially manifold. Then, ρ has a unique non-vanishing eigenstate with eigenvalue one. Thus, $\log ||\rho||_1 = 0$. Following Ref. [13], we can equivalently write

$$\rho = 2^{-N} \sum_{s,r,t} \prod_{v,p,i} \tilde{A}_v^{s_v} \tilde{B}_p^{r_p} \tilde{Z}_v^{t_i}. \quad (25)$$

Next, consider splitting the system into two regions, R and R', and take the partial transpose of ρ on R.

$$\rho^{T_R} = 2^{-N} \sum_{s,r,t} \prod_{v,p,i} \left(\tilde{A}_v^{s_v} \tilde{B}_p^{r_p} \tilde{Z}_v^{t_i} \right)^{T_R} \quad (26)$$

The key to computing the entanglement negativity is to simplify $\left(\tilde{A}_v^{s_v} \tilde{B}_p^{r_p} \tilde{Z}_i^{t_i} \right)^{T_R}$.

In the output state, which is simply the toric code state with product state ancillas, the entanglement negativity can be exactly computed, and has been shown to have a topological correction coming from the long-range topological order [13, 14]. We extend this computation to show the topological correction survives upon performing local Clifford circuits and tracing out the ancillary degrees of freedom.

We start by reviewing the computation in the output, fixed-point state. The first thing to notice is that Z_i trivially commutes with A_v and B_p, because they are supported on different qubits. Second, Z_i is invariant under partial transpose, since the operators are supported on a single site and hence cannot span the boundary between R and R'. Thus, we can pull the Z_i out from the product.

$$\left(A_v^{s_v} B_p^{r_p} Z_i^{t_i} \right)^{T_R} \to \left(A_v^{s_v} B_p^{r_p} \right)^{T_R} Z_i^{t_i}$$

Next, we discuss the effect of partial transpose on the toric code stabilizers, following Ref. [13]. The only stabilizers which are affected non-trivially are those that span the boundary. In particular, if we consider a pair of stabilizers A_v and B_p that intersect at two sites, but only one of the sites is supported in R, then they pick up a minus sign under partial transpose (see Figure S8). More generally, $(A_v^{s_v} B_p^{r_p})^{T_A} = (-1)^{C(s_v,r_p)} A_v^{s_v} B_p^{r_p}$ picks up an overall sign if the number of such intersections $C(s_v, r_p)$ is odd.

We can now discuss the spectrum of the partially transposed density matrix. Note that an orthonormal basis for the Hilbert space can be formed from simultaneous eigenstates of all the stabilizers. These states are labelled by the stabilizer eigenvalues, which can be ± 1. For non-trivial eigenstates of ρ^{T_A}, stabilizers which are unaffected by partial transpose must have eigenvalue one. Thus, we can reduce the spectrum calculation to only stabilizers on the boundary.

Therefore, we can associate eigenstates with expectation values of "strange correlators" of the form $\langle + | \cdot |\psi\rangle$ [13]. Order stabilizers around the boundary, so A_v are on even sites and B_p on odd sites, and consider specifically the correlators,

$$\langle + | \, Z_{2i}^{(1+A_v)/2} Z_{2i+1}^{(1+B_p)/2} \, |\psi\rangle \, .$$

Here, $\psi(s) = (-1)^{\sum_i s_i s_{(i+1)\mathrm{mod} L}}$ is a wavefunction that encodes the signs coming from partial transpose, and $\langle + | = 2^{-L} \sum_s \langle s|$ is a uniform superposition over all configurations. The wavefunction for $\psi(s)$ is the same as the 1D cluster state. Non-vanishing correlators are generated by products of $Z_{2i-1} X_{2i} Z_{2i+1}$ and $Z_{2i} X_{2i+1} Z_{2i+2}$, the stabilizers of the cluster state. There are 2^{L-2} of such non-vanishing correlators, each with expectation value $\pm\langle + | \psi\rangle$. The overlap $\langle + | \psi\rangle$ is 2^{-n+1}, where $L = 2n$. Thus, the entanglement negativity is $S_N = (n-1)\log 2$ where n is the size of the perimeter. The constant correction is a signature of topological entanglement entropy.

Next, we discuss how the effect of finite-depth Clifford circuits on the entanglement negativity. In particular, since Clifford circuits map stabilizers to stabilizers, eigenstates of ρ^{T_A} can still be labelled by stabilizer expectation values, and the entanglement negativity can be calculated by enumerating non-zero strange correlators. However, a few important things change. The set of stabilizers which span the boundary, and hence may have non-trivial twist products with other stabilizers, is considerably larger. Any A_v, B_p, or Z_i stabilizer that is within distance ℓ of the boundary could contribute. Applying the same procedure as in the case of the toric code, we can enumerate non-trivial strange correlators of the form $\langle + | O | \psi' \rangle$ for a different state $\psi'(s)$. However, now the Hilbert space of $\psi'(s)$ has one qubit associated with each relevant A_v, B_p, or Z_i.

Now, we argue that new state $\psi'(s)$ preserves the same cluster-state structure as in the fixed-point case and still has long-range SPT order. First, we show that ψ' still has a $\mathbb{Z}_2 \times \mathbb{Z}_2$ symmetry, generated by the product of Pauli-X operators on all qubits associated with A_v, or all qubits associated with B_p. To see this, notice that the product of all relevant A_v decomposes into two independent Wilson loops, each supported distance ℓ away from the boundary on the interior and exterior sides. Each of these Wilson loops individually has trivial twist-product with the other transformed stabilizers, implying that it can be applied (on either side) to the partially-transposed state ρ^{T_A} without changing the state. This further implies that $\psi(s)$ is invariant under applying the Pauli-X associated with each A_v qubit.

Second, we show that there exists long-range SPT order in $\psi'(s)$. In particular, to do this, we show that there

are coarse-grained stabilizers which have the same behavior under partial transpose as the stabilizers at the toric-code fixed point. Consider large Wilson loops generated by multiplying either A_v or B_p in a large contiguous region spanning the boundary (see Figure S8). In particular, if we look at regions that are offset, the large Wilson loops associated with A_v or B_p necessarily anti-commute on either side of the boundary, leading to a twist product of -1. Therefore, we can construct coarse-grained Pauli-'Z' and 'X' operators in the Hilbert space of ψ' simply by taking products of single-qubit Pauli operators. This argument shows that the coarse-grained ZXZ operator is a stabilizer of the state.

Finally, we discuss the effect of the remaining degrees of freedom on the entanglement negativity. The $\tilde{Z}_i$ stabilizers split into two groups: those that contain support on P, and those that are fully supported on SA. Let us start with the first group. Since we trace out all of subsystem P, the effect on the $\tilde{Z}_i$ stabilizers is to replace the stabilizer state with the maximally mixed state

$$\mathrm{tr}_P\left[\frac{1+\tilde{Z}_i}{2}\right] \to \frac{1}{2}I_i. \tag{27}$$

The operator $I/2$ has two eigenvectors, each with eigenvalue $1/2$. Thus, any stabilizer with support on P becomes fully disentangled from the rest of the system, and has no effect on the entanglement negativity. Next, consider stabilizers that are fully supported on SA. The $\tilde{Z}_i$ which are near the boundary between R and R' will potentially contribute to the entanglement negativity. Furthermore, they may have non-trivial commutation with $\tilde{A}_v$, $\tilde{B}_p$, and other $\tilde{Z}_i$ after partial transposition. Thus, they can contribute to the topological entanglement entropy. However, since only operators near the boundary are affected by partial transpose, these operators only contribute an area-law contribution. Further, we note that they cannot change the long-range SPT order of $\psi(s)$, since they commute with the $\mathbb{Z}_2 \times \mathbb{Z}_2$ symmetry. As such, they also should not affect the value of the topological term.

The argument presented above relied on the fact that the stabilizers of the state were Pauli operators. This meant the spectrum of ρ^{T_A} could be exactly computed, and the techniques of Ref. [13] could be applied. However, the key part of the proof involved generating a sub-algebra of coarse-grained Wilson loops with twist-product -1. LED provides a constructive method for measuring such coarse-grained Wilson loops, with non-trivial twist products, in generic mixed states, as explained in our proof of Theorem ??. Hence, it would be interesting to directly link the existence of operators with non-trivial twist product to the topological entanglement negativity in general mixed states.

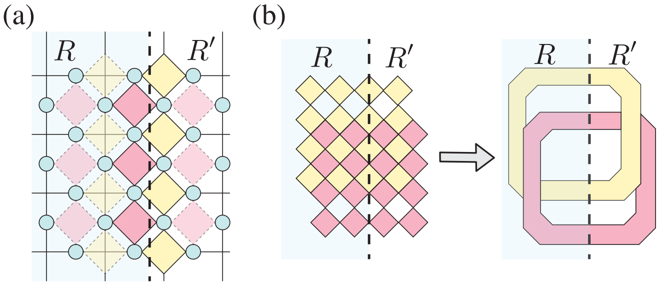

FIG. S8: (a) Calculating entanglement negativity in the toric code state reduces to computing a "strange correlator" in a state that lives on the boundary, where qubits in the state encode stabilizer expectation values. Stabilizers which do not span the boundary do not commute. Hence, they are forced to be $+1$ and do not contribute to the strange correlators. In contrast, vertex and plaquette stabilizers, whose intersections span the boundary pick up -1 signs under partial transpose. This acts like a CZ gate on the boundary state, essentially preparing a cluster state (see text). The long-range SPT order of the cluster state leads to a topological correction to the entanglement negativity. (b) A local (Clifford) unitary circuit coarse-grains each of the stabilizers in the Heisenberg picture. As a result, a much larger number of stabilizers participate in the boundary state. However, we show the long-range SPT order survives, by looking at products of stabilizers. In particular, a product of a large block of stabilizers is supported only on the boundary. After the unitary circuit, this boundary gets coarsened, but for large enough blocks the intersection structure survives. This ensures the operators pick up -1 signs under partial transpose as well.

Valence-Bond Solid Phase

In the main text, we argued that the flow of closed-loop and open-string Wilson operators suggest that the large Δ/Ω regime is most consistent with a decoherence-dominated disorder phase. This in contrast to numerical simulations, which suggest the ground state in this regime is a valance bond solid (VBS) state [15]. The VBS fixed-point state can be understood as a single dimer covering, in contrast to the spin-liquid state which is a uniform superposition over dimer coverings. More generally, in VBS phase the m-anyons become condensed and the e-anyons become confined. As such, in the VBS phase, we would expect that closed Z-loops flow to $+1$, and open Z-strings flow to either $+1$ or -1 depending on how many dimers they intersect. However, our analysis shows that all Z-loops, both open and closed, stay near zero under LED flow. In the main text, we average over all open Z-strings, so the vanishing signal could be due to cancellation of individual loops that flow to opposite values. In Figure S9, we also plot a representative, single Z-string, and see the expectation value still remains close to zero at large Δ/Ω. Since the X-loops also remain at zero, this is the same signature of the high incoherent error regime of the toric code mixed-state phase diagram.

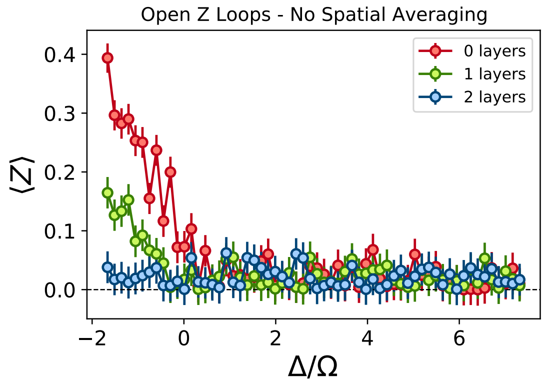

FIG. S9: Flow under LED of a representative, single open Z-string. This string operator remains close to zero in the large Δ/Ω regime, which is inconsistent with preparation of a VBS state.

Detecting Gauge-Glass Phases and More

The LED techniques developed here can also be applied to characterize generalized toric code orders in higher dimensions. The 2D toric code we study has point-like excitations. However, different models can have higher-dimensional excitations, such as line-like excitations appearing in the 2D Ising model and 3D gauge theories. LED can also be applied in these cases. In particular, simple local decoders for models with line-like excitations also exist. For example Toom's rule, as described in Ref. [16], can be made into a local decoder by restricting the number of update steps to some constant. The patch-based decoder can also be generalized to this case by locally identifying small contractible loops. Another strategy would be to consider spatial slices of the model with line-like excitations. This would result in a lower-dimensional model with point-like excitations, for which the techniques presented in this paper can be directly applied.

A particularly interesting case study is the 3D random-plaquette gauge model. The degrees of freedom live on the links of the 3D square lattice. Products of spin operators around each plaquette measure the magnetic flux. Excitations are line-like magnetic flux tubes, coming from chains of excited plaquettes. The model also has quenched disorder, so that the zero-temperature state has a finite density of magnetic flux tubes. Hence, there are two sources of flux tubes, the quenched disorder as well as thermal fluctuations. Due to the gauge constraints, the combination of flux tubes from quenched disorder and thermal fluctuations always form closed loops.

Let K_p be the parameter which determines the density of quenched disorder, and K the effective temperature. The model has three phases of interest to us. The first is an ordered phase for small K and K_p, where the density of flux tubes is small. The second is a disordered phase at large K and K_p, where flux tubes condense. These two phases can be distinguished by measuring the disorder averaged Wilson loop $[\langle W(C)\rangle_K]_{K_p}$. In Ref. [17], it is suggested that these two phases be identified by comparing perimeter-law decay vs. area-law decay in each of the distinct phases. Perimeter-law decay indicates that the fluctuations which cause the Wilson loop to decay are local, and hence characterizes the ordered phase.

The third phase is a disordered but glassy phase at large K_p but small K. In this phase, quenched disorder is large, leading to a high density of flux tubes. As a result, the disorder averaged Wilson loop obeys an area-law, consistent with a disordered phase. However, thermal fluctuations remain small, and the pattern of flux tubes is static when averaged over thermal fluctuations. Therefore, the square of the thermal expectation value, $[\langle W(C)\rangle_K^2]_{K_p}$, should decay as perimeter-law and can hence distinguish the disordered phase from the glassy phase.

The LED observables we construct here can be used to more robustly classify these phases as well. In particular, as explained in the main text, differentiating between perimeter-law and area-law is difficult in practice. Both of these signals decay exponentially in loop size, causing the sample complexity to increase significantly. Further, area-law generically becomes mixed with perimeter-law in the presence of additional imperfections or perturbations, such as incoherent errors or gauge-constraint violating terms. However, using LED, the Wilson loops will be amplified to one only in the ordered phase. Similarly, the squared Wilson loop $\langle W(C)\rangle_K^2$ would be amplified in the ordered and glassy phases. This observable can also be efficiently measured from snapshots, as long as we are able to sample multiple times from each quenched disorder realization. Another strategy, which could be more sample efficient, is the following: Given two independent snapshots s_0, s_1 from the same quenched disorder realization, one can compute the element-wise sum modulo two, $s = s_0 \oplus s_1$, and then measure the LED Wilson loop on the new snapshot s. Intuitively, such a procedure effectively removes the quenched disorder, which is fixed from snapshot-to-snapshot, leaving behind only the thermal fluctuations. Identifying such gauge-glassy models is also difficult in numerical simulations, and hence LED and related ideas could also be applicable in those contexts.

Annulus Decoders

In this section, we consider an alternative approach to constructing error corrected operators, by applying MWPM to an annulus. Anyons supported on the annulus can be paired either with other anyons, or with the boundary of the annulus. Interestingly, MWPM is a global decoder, where introducing or removing anyons

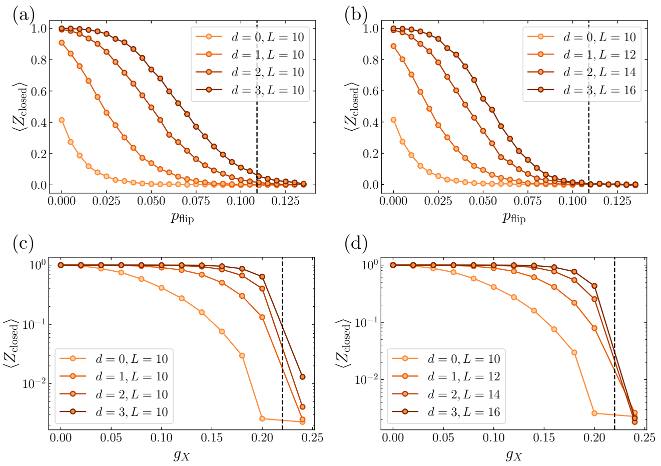

FIG. S10: LED loops using MWPM on an annulus. We see the region classified as topological extends significantly further than the LED loops, for both incoherent (a,b) and coherent (c,d) perturbations. However, additional subtleties arise. When we study fixed loop size and increasing annulus thickness (a,c), there appears to be amplification even on the trivial and disordered sides. However, if we scale the loopsize such that the size of the interior is fixed (b,d), then the improper amplification disappears. An interesting direction for future work is to develop a rigorous understanding of whether non-local annulus-based decorating can also serve as a topological order witness.

can change the pairing far away. However, by construction, the decoder cannot connect the interior of the annulus to the exterior.

We show in Figure S10 the behavior of these corrected loop operators for coherent, incoherent, and mixed errors. In particular, for the incoherent error model, we see the region classified as topological seems to coincide with the known error recovery threshold of $\approx 10.1\%$ for MWPM. This is much closer to the theoretical optimal error correction threshold of $\approx 10.9\%$, where we expect the phase transition from topological to disordered to occur.

Since this decoder is non-local, our existing theoretical arguments, and the connection to RG, may not apply. Nevertheless, an annulus decoder cannot change the super-selection sector—whether or not an anyon is contained within the annulus. Therefore, if the annulus-correction decorated loops go to one, then asymptotically large regions have well-defined super-selection sectors, suggesting the state is topologically-ordered. Indeed, it has been argued that such an annulus decoder could be considered a witness for topological entanglement entropy [18].

SUPPLEMENTARY NOTES

Definition and Properties of Fixed-Point States

As discussed in the main text, one important component for defining any LED procedure is to identify a fixed-point state of the target phase of matter. Here, we review the definition and key properties of a fixed-point state.

It is well-known in the literature that gapped quantum ground states can be classified by a real-space RG flow [8, 20]. To implement such an RG flow, one notes that any two states in the same phase can be connected by finite-depth local unitary transformations. The hallmark property of topologically-ordered states is the presence of long-range entanglement, and finite-depth local unitary transformations can add or remove local short-range entanglement; thus, for any given state in a topological phase of matter, one can construct a procedure which hierarchically removes all short-range entanglement from this state at increasing length-scales. Then, the resulting state then has zero correlation length, as all short-range entanglement has been removed; this state is known as a fixed-point state of the topological phase.

We also consider important properties of the fixed-point state in the context of our LED procedure and the closely related QCNN procedure of Ref. [19]. In both of these procedures, a fixed-point state of the phase under consideration is chosen, and the protocol identifies states within this phase by removing local errors or perturbations on top of this fixed-point state; this is done by performing a decoding operation and a coarse-graining operation, and repeating them n times. In these settings, the fixed-point state has a few special properties: If the input state is equal to the fixed-point state, no errors are detected within each decoding step, and furthermore, the state after each layer of decoding and coarse-graining is equal to the input state, defined on a subsystem of the original system with fewer qubits. Finally, because fixed-point states have zero correlation length, the bare Wilson loops defined in the main text have expectation value equal to $+1$ without performing any correction or LED.

Background on Generic Topological Phases

Here, we provide background on generic topological quantum field theories (TQFTs), which are characterized by modular tensor categories $\mathcal{C}$. The possible topological charges (a.k.a. anyon types) in such a system are given by the simple objects $\{\alpha_0, \alpha_1, \alpha_2, ...\}$ of $\mathcal{C}$, where $\alpha_0 = \mathbf{1}$ is the trivial or vacuum topological charge. Abelian anyons α_i have quantum dimension $d_i = 1$, meaning that the outcome of fusing α_i with any other anyon α_j is deterministic: $\alpha_i \otimes \alpha_j = \alpha_{k(i,j)}$ for some integer $k(i,j)$. On the other hand, non-abelian anyons α_i, α_j have quantum di-

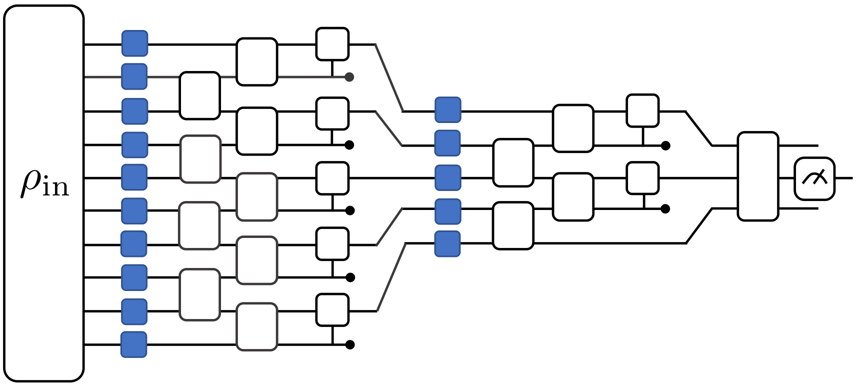

FIG. S11: One-dimensional illustration of a hierarchical LED circuit, inspired by the QCNN circuit of Ref. [19]. Here, stabilizer measurements are performed at each LED layer using local unitary gates (white boxes), and the LED error-correction step is performed through controlled-unitary gates. While this particular circuit model maps stabilizer values in each LED layer to qubits in the initial system ($\rho_{\rm in}$) which are measured in that layer, the stabilizer values can also be obtained by introducing ancillary qubits in a known state (e.g $|0\rangle$) and performing local gates in the same fashion as for surface-code quantum computation [3]. To accommodate states which differ from a known, fixed-point state by local rotations, variational unitary operations (blue boxes) can be introduced before each LED stabilizer-measurement step, and the parameters to these unitaries can be optimized adaptively through a hybrid quantum-classical feedback loop to achieve high LED operator expectation values.

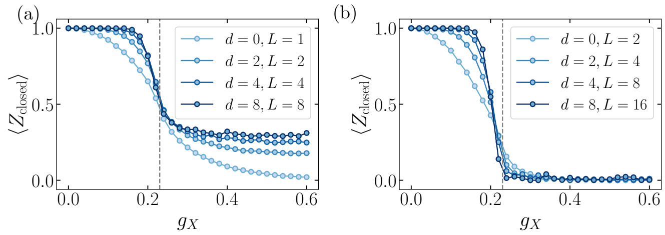

FIG. S12: Over-correction for small Wilson loops. (a) Small LED loops with $d > L$ can give non-zero signal deep in the trivial phase, as in this regime, correction can pump anyons from the interior of the annulus to the exterior. (b) However, once $d < L$, the effects of over-correction become insignificant. LED Wilson loops are theoretically expected to certify topological order in the regime of $d \ll L$.

mensions $d_i, d_j > 1$, and their fusion can result multiple possible anyon types as governed by *fusion rules*

$$\alpha_i \otimes \alpha_j = \oplus_k n_{ij}^k \alpha_k. \tag{28}$$

Here, the fusion coefficients n_{ij}^k must satisfy

$$d_i d_j = \sum_k n_{ij}^k d_k. \tag{29}$$

Moreover, each anyon α_i has a unique *conjugate* anyon $\alpha_j = \overline{\alpha_i}$ for which $n_{ij}^{\mathbf{1}} = 1$ (i.e., α_i and $\overline{\alpha_i}$ can annihilate each other by fusing to vacuum); for all other α_k, $n_{ik}^{\mathbf{1}} = 0$.

In addition to anyon types and fusion rules, several other quantities are needed to characterize a TQFT. In particular, for every pair of anyons α_i, α_j, one can compute the *Hopf link* anyon braiding statistics:

$$\tilde{s}_{ij} = \tag{30}$$

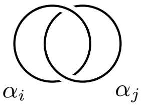

Moreover, for each anyon α_i, its *topological twist* is defined as

$$\theta_i = \frac{1}{d_i} \tag{31}$$

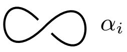

These quantities are used to define the modular S and T matrices of $\mathcal{C}$:

$$S_{ij} = \frac{1}{\mathcal{D}} \tilde{s}_{ij}, \qquad T_{ij} = \delta_{ij} \theta_i. \tag{32}$$

where $\mathcal{D} = \sqrt{\sum_i d_i^2}$ is the global quantum dimension of $\mathcal{C}$.

It is conjectured that the modular S and T matrices uniquely define a unitary modular tensor category, or equivalently a topological phase of matter [21]. In the main text and Methods, we illustrate the application of LED to Levin and Wen's string-net models based on arbitrary unitary fusion categories $\mathcal{A}$. The MTC describing such a string-net model is the Drinfeld center $\mathcal{C} = \mathcal{Z}(\mathcal{A})$ [22]. Additional background on general TQFTs and MTCs can be found in Refs. [21, 23].

[1] M. Iqbal and N. Schuch, "Entanglement Order Parameters and Critical Behavior for Topological Phase Transitions and Beyond," Physical Review X **11**, 041014 (2021).

[2] K. Duivenvoorden, M. Iqbal, J. Haegeman, F. Verstraete, and N. Schuch, "Entanglement phases as holographic duals of anyon condensates," Phys. Rev. B **95**, 235119 (2017).
[3] A. G. Fowler, M. Mariantoni, J. M. Martinis, and A. N. Cleland, "Surface codes: Towards practical large-scale quantum computation," Phys. Rev. A **86**, 032324 (2012).
[4] M. Aguado and G. Vidal, "Entanglement renormalization and topological order," Phys. Rev. Lett. **100**, 070404 (2008).
[5] A. Kitaev, "Fault-tolerant quantum computation by anyons," Annals of Physics **303**, 2–30 (2003), publisher: Elsevier BV.
[6] M. B. Hastings and X.-G. Wen, "Quasiadiabatic continuation of quantum states: The stability of topological ground-state degeneracy and emergent gauge invariance," Phys. Rev. B **72**, 045141 (2005), publisher: American Physical Society.
[7] M. Levin and X.-G. Wen, "Detecting Topological Order in a Ground State Wave Function," Phys. Rev. Lett. **96**, 110405 (2006), publisher: American Physical Society.
[8] X. Chen, Z.-C. Gu, and X.-G. Wen, "Local unitary transformation, long-range quantum entanglement, wave function renormalization, and topological order," Physical Review B **82**, 155138 (2010).
[9] M. A. Nielsen and I. L. Chuang, *Quantum Computation and Quantum Information: 10th Anniversary Edition*.
[10] J. Haah, "An invariant of topologically ordered states under local unitary transformations," Communications in Mathematical Physics **342**, 771–801 (2016), publisher: Springer.
[11] A. Peres, "Separability Criterion for Density Matrices," Physical Review Letters **77**, 1413–1415 (1996).
[12] M. Horodecki, P. Horodecki, and R. Horodecki, "Separability of mixed states: necessary and sufficient conditions," Physics Letters A **223**, 1–8 (1996).
[13] T.-C. Lu and S. Vijay, "Characterizing long-range entanglement in a mixed state through an emergent order on the entangling surface," (2022), 10.48550/ARXIV.2201.07792.
[14] Y. A. Lee and G. Vidal, "Entanglement negativity and topological order," Physical Review A **88**, 042318 (2013).
[15] R. Verresen, M. D. Lukin, and A. Vishwanath, "Prediction of toric code topological order from Rydberg blockade," Phys. Rev. X **11**, 031005 (2021).
[16] N. P. Breuckmann, K. Duivenvoorden, D. Michels, and B. M. Terhal, "Local decoders for the 2d and 4d toric code," (2016), 10.48550/ARXIV.1609.00510.
[17] C. Wang, J. Harrington, and J. Preskill, "Confinement-higgs transition in a disordered gauge theory and the accuracy threshold for quantum memory," Annals of Physics **303**, 31–58 (2003).
[18] J. R. Wootton, "A witness for topological order and stable quantum memories in abelian anyonic systems," Journal of Physics A: Mathematical and Theoretical **45**, 395301 (2012).
[19] I. Cong, S. Choi, and M. D. Lukin, "Quantum convolutional neural networks," Nature Physics **15**, 1273–1278 (2019), publisher: Nature Publishing Group.
[20] N. Schuch, D. Pérez-García, and I. Cirac, "Classifying quantum phases using matrix product states and projected entangled pair states," Phys. Rev. B **84**, 165139 (2011).
[21] Z. Wang, *Topological quantum computation*, 112 (American Mathematical Soc., 2010).
[22] M. A. Levin and X.-G. Wen, "String-net condensation: A physical mechanism for topological phases," Phys. Rev. B **71**, 045110 (2005).
[23] B. Bakalov and A. A. Kirillov, *Lectures on tensor categories and modular functors*, Vol. 21 (American Mathematical Soc., 2001).
